# Better the devil you know: sympatric fish host copes better than allopatric with a myxozoan parasite

**DOI:** 10.3389/fcimb.2025.1576014

**Published:** 2025-06-27

**Authors:** Itziar Estensoro, Beatriz López-Gurillo, Carolina Tafalla, Ryan Craig, Stephen Atkinson, Ariadna Sitjà-Bobadilla, Jerri Bartholomew

**Affiliations:** ^1^ Fish Pathology Group, Instituto de Acuicultura Torre de la Sal, Consejo Superior de Investigaciones Científicas (IATS, CSIC), Castellón, Spain; ^2^ Biotechnology Department, Instituto Nacional de Investigación y Tecnología Agraria y Alimentaria (INIA), Consejo Superior de Investigaciones Científicas (CSIC), Madrid, Spain; ^3^ Department of Microbiology, Oregon State University, Corvallis, OR, United States

**Keywords:** *Ceratonova shasta*, genotype 0, intestinal inflammatory response, steelhead trout strains, lymphocytes, specific IgM, IgT, IgD

## Abstract

Ceratomyxosis due to the myxozoan parasite *Ceratonova shasta* affects salmonids, causing severe enteritis leading to hemorrhaging and necrosis. The waterborne parasite stages penetrate host gills and reach the fish intestine through the bloodstream. Steelhead trout (*Oncorhynchus mykiss*) populations from *C. shasta* endemic watersheds (sympatric) are less susceptible than populations from non-endemic watersheds (allopatric). We investigated the immune effectors behind these different susceptibilities. Both steelhead trout strains were exposed to *C. shasta* genotype 0. Intestinal tissue and serum samples of control and exposed sympatric and allopatric fish were taken at 8, 15, 22, 29, 57, and 183 days post exposure. Gills were taken at 1 and 8 days post exposure. Parasite abundance and histopathology were evaluated on tissue sections of fish that tested PCR+ for the parasite. Zap70^+^ T cells, IgT^+^ B cells, and IgD^+^ B cells were quantified, and the presence of specific IgM was evaluated from fish sera by immunohistochemistry. Parasite counts were significantly lower and limited to a shorter duration in sympatric fish. The initial intestinal inflammatory response in both fish strains was characterized by hyperplasia of the lamina propria–submucosa and epithelial infiltration of lymphocytes. Remarkably, hyperplasia was resolved earlier in sympatric fish, whereas in allopatric fish, hyperplasia was not resolved by the end of the experiment, coinciding with high intestinal parasite counts and sporogenesis. An increase of intestinal Zap70^+^ T cells occurred before IgT^+^ B cells peaked, earlier in sympatric than in allopatric fish. Low numbers of intestinal IgD^+^ B cells were detected in both strains. In gills, an early increase of Zap70^+^ T cells was observed in both fish strains at 1 day post exposure, and an increase of IgD^+^ B cells occurred only in the allopatric strain. Specific circulating IgM was detected much later in both fish strains, only at 57 and 183 days post exposure. In conclusion, sympatric steelhead trout restricted intestinal proliferation of *C. shasta* genotype 0 more effectively than allopatric fish, probably due to an earlier T cell response triggering a stronger IgT-based mucosal adaptive response in the intestine. Specific circulating IgM appeared later in both sympatric and allopatric fish, probably also contributing systemic protection.

## Graphical Abstract

Overview of infection and response kinetics of sympatric (SYM, green) and allopatric (ALLO, yellow) steelhead trout exposed to *Ceratonova shasta* G0, during a 183-day experimental parasite challenge. Red triangles pointing up represent increase of a given parameter. ▲= increase.

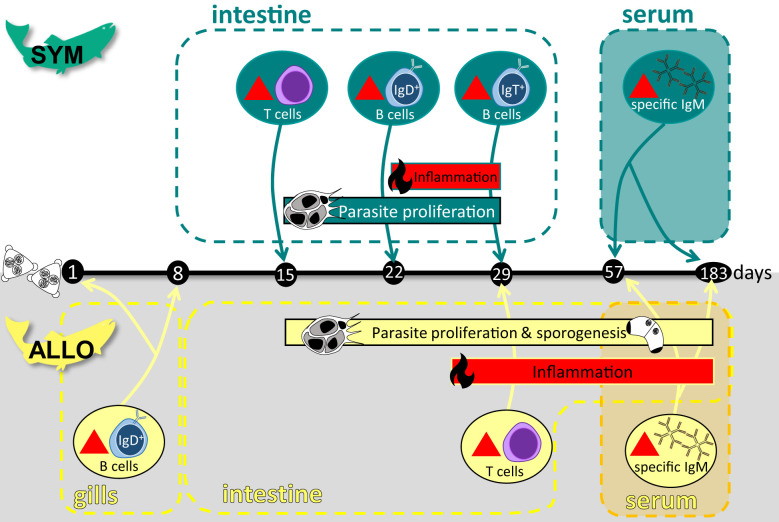

## 1 Introduction

The myxozoan parasite *Ceratonova shasta* is endemic across the Pacific Northwest of the U.S. and Canada, and comprises at least three genotypes that affect different species of salmon and trout (Atkinson and Bartholomew, 2010b, a; Stinson and Bartholomew, 2012). Much of the research on *C. shasta* host–parasite interactions has focused on the more virulent genotypes, which affect endangered and threatened salmon populations: genotype I (GI), with specificity for chinook salmon (*Oncorhynchus tshawytscha*) (Hurst and Bartholomew, 2012; Bjork et al., 2014), and genotype II (GII), which has a broader host range with coho salmon (*Oncorhynchus kisutch*) as the primary host (Barrett and Bartholomew, 2021; Barrett et al., 2021; Taggart-Murphy et al., 2021).

Genotype 0 (G0) causes non-lethal infections in rainbow trout/anadromous steelhead trout (*O. mykiss*) exclusively. G0 infections are rarely documented in *O. mykiss* native to waters where the parasite is endemic (sympatric). The ability of rainbow trout native to rivers where *C. shasta* is absent (allopatric) to survive G0 infections supports the low virulence of this genotype (Atkinson and Bartholomew, 2010b). In contrast, allopatric salmonid strains are highly susceptible to infections by GI (chinook salmon) and GII (coho salmon, rainbow trout) and have served as infection models because of the low dose required to cause disease and mortality (Atkinson and Bartholomew, 2010a, b; Bjork and Bartholomew, 2010; Hurst and Bartholomew, 2012; Bjork et al., 2014).

Because of its low virulence, G0 infections have received less attention than GI and GII. However, there are several aspects of this genotype that make it unique and a compelling target for research. Evolutionarily, the ancestral host–parasite relationship for *C. shasta* was likely *O. mykiss* and G0 (Breyta et al., 2020), and understanding this relationship may provide insight into how these hosts and parasites co-exist. In contrast to the acute and lethal infections observed with GI and GII infections, G0 causes a chronic infection that culminates in long-term proliferation of the parasite in the intestinal lumen (Taggart-Murphy et al., 2021). This suggests a mode of transmission that would allow continuous parasite dissemination throughout the migratory range of the host, in contrast to a transmission model for GI and GII that relies largely on the death of the fish host, and therefore a point source dissemination (Bartholomew et al., 2022). This also suggests that *O. mykiss* has evolved parasite-specific defenses, and identification of these traits could inform selection of *C. shasta*-resistant fish strains and enhancement of protective host responses.

To better understand how *C. shasta* G0 interacts with *O. mykiss*, we challenged a strain of steelhead trout sympatric with *C. shasta* (i.e., adapted host) and an allopatric strain (i.e., non-adapted “susceptible” host) with a known parasite dose in the laboratory. We characterized host–parasite dynamics in these adapted and non-adapted hosts focusing on the parasite distribution and proliferation kinetics and on the host immune response at the portal of entry, the gills, and the main parasite target, the intestine. Histopathological inflammatory alterations and central immune effectors involved in the mounting of the adaptive immune response were studied. Among the cellular effectors, we analyzed activated T cells, which mediate B cell activation and antibody production upon specific antigen recognition (Laing and Hansen, 2011), and B cells, responsible for the specific immune response. We focused our B cell analysis on the IgT^+^ subset, since IgT is the teleost-exclusive immunoglobulin isotype, specialized in mucosal immunity, and on the IgD^+^ subset, since although little is known about its tissue distribution and dynamics upon infection (Holzer et al., 2021), IgD has also been associated with mucosal responses in fish (Castro et al., 2014; Perdiguero et al., 2019; Herranz-Jusdado et al., 2023). Generally, IgM^+^ B cells are most abundant in hematopoietic organs and blood of teleosts, although they are also present in mucosal tissues. Therefore, soluble IgM prevails in blood and was evaluated here in the steelhead sera.

## 2 Materials and methods

### 2.1 Fish

Strains of steelhead were obtained from hatcheries in Oregon, USA, where *C. shasta* is endemic (sympatric) or absent (allopatric): allopatric winter steelhead from Alsea Hatchery (44°25′N, 123°33′W; ALLO; allopatric strain) and sympatric summer steelhead from Round Butte Hatchery (44°60′N, 121°27′W; SYM; sympatric strain). Fish were transported in coolers with aerated, UV-sterilized well water ~13°C and held at the Oregon State University (OSU) John L. Fryer Aquatic Animal Health Laboratory (AAHL) in 380-L circular tanks on flow-through UV-sterilized well water. At the start of exposure, ALLO fish had an average weight of 23.8 g and a fork length of 13.7 cm; SYM fish had an average weight of 31.5 g and a fork length of 14.3 cm.

### 2.2 Infection of annelid host and harvest of G0 for fish exposures

Populations of the annelid host *Manayunkia occidentalis* were collected at two sites in the Klamath River, CA, USA (42°07’03.3”N 122°03’03.8”W and 41°51’35.6”N 122°34’00.3”W) (Alexander et al., 2016). Annelids were held at the AAHL in two, 60-L mesocosm tanks partially filled with gravel/cobble substrate and supplied with flow-through, UV-sterilized Willamette River water as a nutrition source. The annelids were held for 12 months to purge any existing parasite. During this time, effluent was sampled weekly (1 L), filtered (at 5 µm), and analyzed by qPCR (Hallett and Bartholomew, 2006). Once samples of effluent consistently tested negative for *C. shasta*, annelids were inoculated with *C. shasta* myxospores from intestines and fecal matter of rainbow trout infected with G0 by exposure in the Klamath River (Alexander et al., 2016). Prior to adding myxospores to the mesocosm, a sample was sequenced (Atkinson et al., 2018) to confirm that only G0 was present. Annelids were dosed with G0 myxospores weekly for 12 wk. During this time, samples of effluent were collected weekly, following a 24-h static period, and analyzed by qPCR to quantify G0 actinospore production.

Seeding of mesocosms with infected tissue was ceased 10 d prior to fish exposure to determine infectious dose in water samples and minimize contribution from myxospore stages. For a week prior to exposure, mesocosms were maintained in static conditions with aeration. Approximately 10 L of water was removed from each mesocosm every 24 h, and the tanks were refilled with UV sterilized Willamette River water. For the exposure, ~130 L of water harvested from the mesocosms were stored in an aerated, 190-L polyethylene container. Parasite spores were up to 1 wk old at the time of exposure to fish. Three 1-L samples analyzed by qPCR measured an average density of 75 C*. shasta* actinospores/L.

### 2.3 Fish infection

Fish exposures were randomized across 16 100-L tanks, with 4 tanks for exposure of each steelhead strain and 4 tanks containing controls of each strain. 130 L of harvested mesocosm water (containing 75 C*. shasta* actinospores/L) was divided into 8 aerated exposure tanks (16.5 L per tank; ~1238 actinospores/tank). An equivalent amount of 13°C UV-sterilized well water was added to 8 aerated control tanks. 104 fish from each strain were randomly assigned to exposure and control tanks with 4 replicates of 16 fish exposed per strain (~78 actinospores/fish) and 4 replicates of 10 control fish per strain. Fish were held in the aerated bath exposures for 24 h, after which 13°C UV-sterilized well water flow was resumed. Temperature of flow through water was gradually increased to 18°C over 3 d. Fish were held for 183 d, fed daily, and monitored for disease signs and mortality.

### 2.4 Sample collection

Sampling of exposed and control fish occurred on 1, 8, 15, 22, 29, 57, and 183 days post-exposure (dpe). One fish from each control group and two fish from each exposure group were selected randomly and killed using MS222 (0.1 g/L; Sigma-Aldrich). Blood was drawn from the caudal vessels with a heparinized syringe, and transferred to 2-mL tubes. The blood was refrigerated overnight and then centrifuged at 3000 × *g* for 5 min. Serum was drawn off using a pipette, transferred to 0.5-mL vials, and frozen at −20°C for determination of specific antibodies.

Fish were dissected immediately after blood collection, and 3 transverse sections of intestine were removed. The first (posterior) and third (anterior) sections were cut to ~0.5 cm and placed into a 2-mL tube containing RNAlater, refrigerated overnight, and then divided into 2 × 2-mL tubes and frozen—one tube for transcriptome analysis, the other for qPCR. The second, middle section of intestine, was cut to 2–3 cm and placed in buffered formalin for 48 h before transfer to 70% ethanol for histology. Tools used for dissections were soaked in H_2_O_2_ for 3–5 min and then rinsed in deionized water between samples.

At 1 and 8 dpe, two gill arches were removed from each fish. One was placed in buffered formalin for 48 h before transfer to 70% ethanol for histology; the other was placed in a 2-mL tube and frozen for qPCR.

For mortalities that occurred during the study, skin scrapings and intestinal swabs were examined microscopically for external parasites and presence of *C. shasta* myxospores, respectively. Tissue samples of the intestine were taken for PCR assay.

### 2.5 PCR and qPCR

Fish intestinal tissue samples were digested using a modified “boiled-crude” method of Palenzuela et al. (1999): incubation at 56°C for 1–2 h with 195 μL buffer ATL (Qiagen) and 5 μL proteinase K (20 mg/mL) to digest tissue, followed by heat denaturation at 85°C for 15 min, and then dilution 1:100 prior to assay by PCR (Cs1479F GCATCACCTGCTCTGAGAAGAGTGG, Cs2067R GGTCTTCATCGATGTTTTTGCCGAGG) (Atkinson and Bartholomew, 2010a; Atkinson et al., 2018). Parasite genotype was determined by sequencing PCR products using an ABI BigDye Terminator Cycle Sequencing Kit v3.1 and ABI3730 Genetic Analyzer (Applied Biosystems, Foster City, CA, USA) at the Center for Quantitative Life Sciences, CQLS, OSU (Atkinson and Bartholomew, 2010b). Fish gill samples were processed using the Qiagen DNeasy Animal Tissue Kit (Qiagen) using the manufacturer’s instructions. qPCR was used to determine presence and amount of parasite in gill samples and in 1-L water samples, and was performed using a modified *C. shasta* SSU rDNA qPCR assay (Cs1034F CCAGCTTGAGATTAGCTCGGTAA, Cs1104R CCCCGGAACCCGAAAG, TaqMan Probe Cs1058T 6FAM-CGAGCCAAGTTGGTCTCTCCGTGAAAAC-TAMRA) (Hallett et al., 2012; Alama-Bermejo et al., 2019). For gill tissue samples, we used the same DNA amount per reaction (134 ng) and ran a four-point 10-fold dilution standard curve of a purified PCR product to calculate SSU rDNA copy numbers of the parasite. All samples were run in triplicate, with a positive *C. shasta* sample as an interplate calibrator, and a no-template control. The corresponding formalin-fixed samples of fish tissue that tested positive for *C. shasta* DNA by PCR or qPCR were selected for histopathological assessment.

### 2.6 Histopathological analysis

Formalin-fixed gill and intestine samples were routinely dehydrated and processed for paraffin histology. One gill and two intestinal cross-sections were cut at 4 µm, stained with Giemsa, and then used to evaluate parasite prevalence and intensity of infection. Intensity was scored from 1 to 6 based on the number of parasite stages observed in the entire tissue section: 1 = 1–25 parasite stages; 2 = 26–50 parasite stages; 3 = 51–100 parasite stages; 4 = 101–150 parasite stages; 5 = 151–200 parasite stages; 6 = >200 parasite stages. In intestines, proliferative trophozoites that infiltrated into the lamina propria–submucosa or epithelium, free luminal proliferative trophozoites, and free luminal disporoblasts were scored separately.

Intestinal thickness was measured from the outer *muscularis* layer to the epithelium in intestinal cross-sections, including *muscularis* layers, *stratum granulosum*, *stratum compactum*, and lamina propria–submucosa. Intestinal hyperplasia was evaluated by measuring the surface area of the lamina propria–submucosa in intestine cross-sections. Lymphocyte epithelial infiltration in the intestine was scored ranging from 0 (absence) to 3 (very abundant: 25–30 intraepithelial lymphocytes [IELs] per microscope field at 500× magnification).

Observations were performed with a Leitz Dialux22 (Leica, Hesse, Germany) light microscope, and images were taken with an Olympus DP70 Camera (Olympus, Tokyo, Japan). Thickness and surface measurements were performed with ImageJ.

### 2.7 Lymphocyte immunohistochemistry

Paraffin-embedded tissue sections were collected on Superfrost Plus slides (Menzel-Gläser, Braunschweig, Germany) for immunolabeling of lymphocytes: Zap70+ T cells, IgT^+^ B cells, and IgD^+^ B cells were analyzed. Details on antibodies are included in **Table 1**.

**Table 1:** Primary antibodies used for immunolabeling of trout lymphocytes (*) and parasite stages.

Primary antibody	Origin	Producing species	Concentration (µg/mL)	Epitope retrieval buffer	Incubation conditions
Anti-human Zap70 *	Cell signaling	Rabbit	1:50^§^	Sodium citrate, pH 6	ON, 4°C
Anti-trout IgT *	Ballesteros et al. (2013)	Mouse	3	Tris-EDTA, pH 9	ON, 4°C
Anti-trout IgD *	Ramírez-Gómez et al. (2012)	Mouse	5	Tris-EDTA, pH 9	1 h, RT
Anti-trout IgM	Ballesteros et al. (2013)	Mouse	10	Tris-EDTA, pH 9	1 h, RT

ON, overnight; RT, room temperature; ^§^manufacturer’s instructions.

Washing steps between incubations consisted of successive 5-min immersions in TTBS (20 mM Tris-HCl, 0.5 M NaCl, 0.05% Tween 20, pH 7.2) and TBS (20 mM Tris–HCl, 0.5 M NaCl, pH 7.2), and incubations were made in a humid chamber at room temperature unless otherwise stated. Tissue sections were routinely deparaffinized and hydrated, before endogenous peroxidase was quenched in 0.3% H_2_O_2_ for 30 min. Heat-induced epitope retrieval was carried out by submerging the slides in the corresponding buffer solution and then heating them in a microwave oven for 5 min at 800 W and 5 min at 450 W. Tissues were incubated with primary antibodies diluted in 1% BSA in TBS, then with the corresponding biotinylated secondary antibody (horse anti-mouse or goat anti-rabbit), and then with avidin–biotin–peroxidase complex (ABC) following the manufacturer’s instruction (Vector Laboratories, CA, USA). Finally, bound peroxidase was visualized after 2 min incubation with 3,3′-diaminobenzidine tetrahydrochloride chromogen (DAB, Sigma-Aldrich, MO, USA), and the reaction was stopped with deionized water. Slides were counterstained with Gill’s hematoxylin, dehydrated, and mounted with di-N-butyl phthalate in xylene. Negative controls omitted the primary antibodies, secondary antibody, and ABC. Zap70^+^, IgT^+^, and IgD^+^ immunoreactive lymphocytes were counted in 10 random microscope fields at ×500 magnification for each fish (2 control ALLO, 2 control SYM, 4 exposed ALLO, and 4 exposed SYM), and means were calculated for each fish and tissue.

Specific circulating IgM against *C. shasta* in the sera of the ALLO and SYM fish was detected immunohistochemically on parasite stages of intestinal sections with high infection intensity (score 6) in an indirect immunohistochemical sandwich ELISA. Briefly, endogenous peroxidase was quenched in tissue sections as described above. Sections were then incubated with pooled serum samples (*N* = 4) of each time point and strain for exposed fish, and 1 unexposed control serum pool (*N* = 4) of each strain. 16 serum pools (7 exposure times × 2 fish strains and 1 unexposed control × 2 fish strains) were tested, diluted 1:25 in 1% BSA. Parasite-bound specific Ig of sera was immunolabeled as described above, i.e., sections were incubated with mouse anti-trout IgM or IgT antibodies, horse anti-mouse biotinylated antibody, ABC, and DAB, and slides were then counterstained and mounted. The presence and intensity of immunoreactivity in the different parasite stages were evaluated, and representative images were taken.

### 2.8 Transmission electron microscopy

Four intestine samples of infected ALLO steelhead trout were fixed immediately in 2.5% glutaraldehyde in 0.1 M sodium cacodylate buffer at pH 7.2 for 24 h at 4°C and washed in the same buffer. Post-fixation was carried out for 1 h in 1% osmium tetroxide in the same buffer, and samples were dehydrated and embedded in Spurr’s resin. Ultrathin sections were contrasted in uranyl acetate and viewed using a Hitachi HT7800 transmission electron microscope (TEM) operating at an accelerating voltage of 120 kV.

### 2.9 Statistics

Data on the observed intestinal histopathological alterations (intestinal thickness, hyperplasia, and IEL scoring) and lymphocyte counts in gills and intestines (Zap70^+^ T cells, IgT^+^ B cells, and IgD^+^ B cells) were analyzed for statistical significance. First, Student *t*-tests were performed to compare SYM vs. ALLO within the control unexposed experimental fish. Since no differences were found (**Supplementary Table 1**), these data were pooled into a single control unexposed experimental group for the subsequent analyses. Secondly, differences were analyzed between data of control unexposed, exposed SYM, and exposed ALLO experimental groups within each time point by one-way analysis of variance (ANOVA-I) followed by Student–Newman–Keuls test, and for intestines also along the six time points. Data that failed the normality or equal variance test were analyzed with Kruskal–Wallis ANOVA-I on ranks followed by Dunn’s method. Data on lymphocyte counts in gills were compared by Student *t*-test or by Mann–Whitney *U* sum test for non-normally distributed data. In addition, data of the different intestinal alterations and lymphocyte counts were pooled regardless of the infection time for the three experimental groups and compared by ANOVA-I followed by Student–Newman–Keuls test, or by Kruskal–Wallis ANOVA-I on ranks followed by Dunn’s method for non-normal distributed data.

Data on parasite intestinal infection intensity for the different parasite stages (intratissue proliferative trophozoites, luminal proliferative trophozoites, and luminal disporoblasts) were analyzed for differences between SYM and ALLO fish within each time point by Student *t*-test or by Mann–Whitney *U* sum test for non-normal distributed data, and along the six time points by ANOVA-I followed by Student–Newman–Keuls test, or by Kruskal–Wallis ANOVA-I on ranks followed by Dunn’s method for non-normal distributed data. SigmaPlot v14.5 software (Systat Software Inc., CA, USA) was used for statistical analyses, and the significance level was set at *P* < 0.05.

### 2.10 Ethical approval

Fish exposures were conducted at the AAHL under Animal Care and Use (ACUP) permit #21-0141.

## 3 Results

### 3.1 Infection outcome: molecular detection and intestinal parasite counts

**Figure 1:**
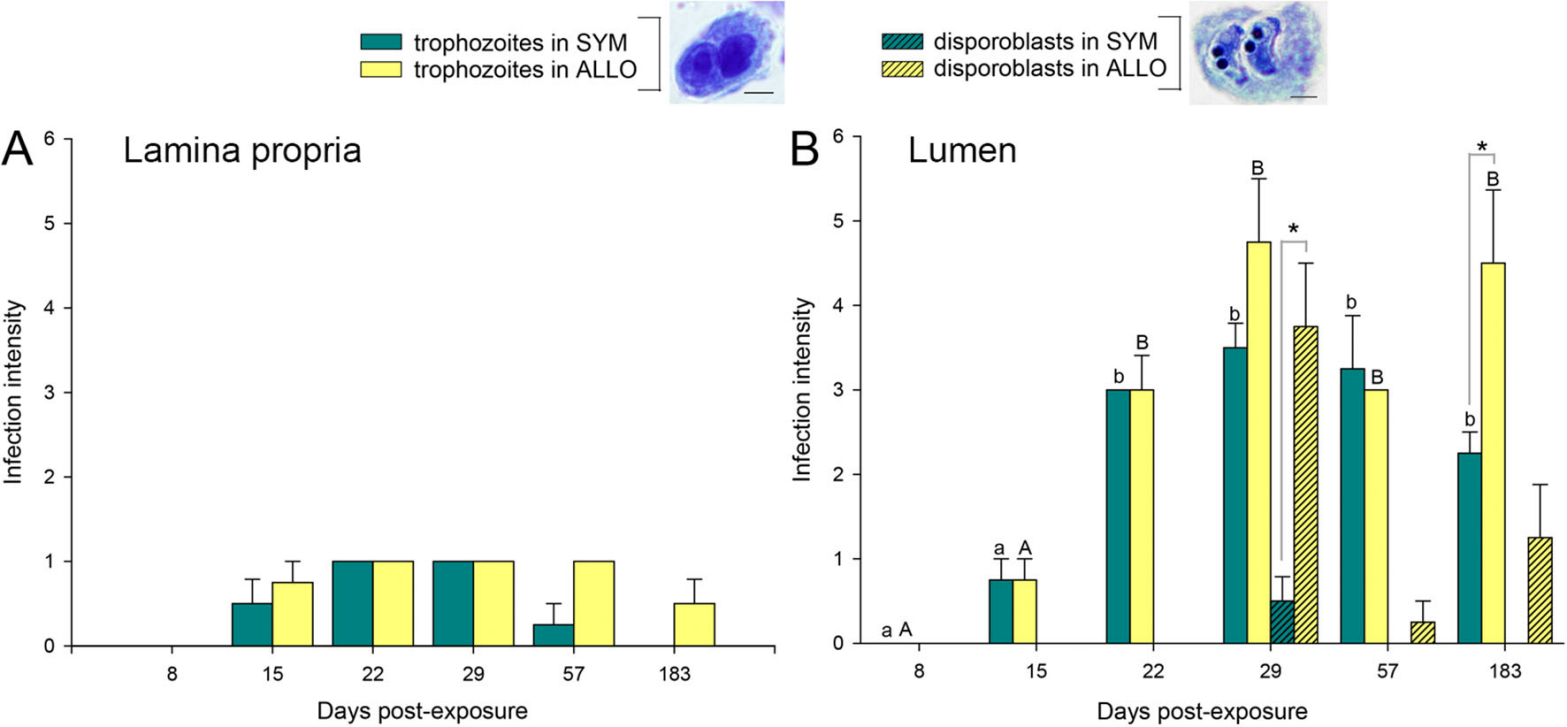
*Ceratonova shasta* infection intensity in exposed sympatric (SYM) and allopatric (ALLO) steelhead trout intestines along the parasite challenge. **(A)** Proliferative trophozoites infiltrated in the lamina propria–submucosa or epithelium. **(B)** Free luminal parasite stages: proliferative trophozoites and disporoblasts. Infection intensity score is the number of parasite stages observed in the entire intestinal cross-section: 1 = 1–25; 2 = 26–50; 3 = 51–100; 4 = 101–150; 5 = 151–200; 6 = 200–300. Lowercase and uppercase letters indicate significant differences among sampling times within SYM or ALLO fish, respectively. Asterisks indicate significant differences between SYM and ALLO fish within a time post-exposure. *P* < 0.05. Scale bars = 20 µm.

**Figure 2:**
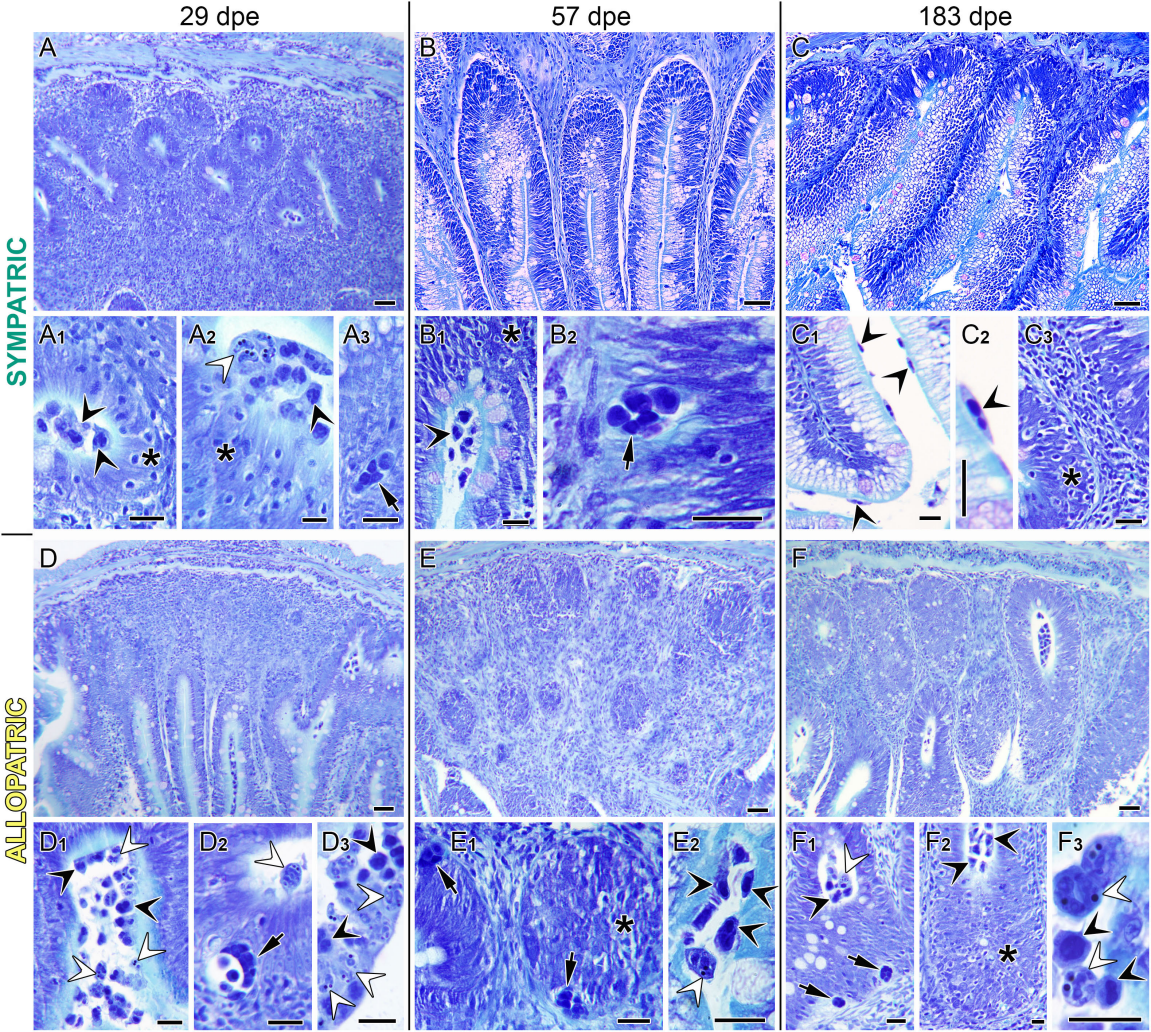
Intestinal micrographs of exposed sympatric (SYM; **A–C**) and allopatric (ALLO; **D–F**) steelhead trout at 29 **(A, D)**, 57 **(B, E),** and 183 **(C, F)** days post-exposure (dpe). In SYM fish, note the severe submucosal hyperplasia at 29 dpe **(A)**, which subsided at 57 **(B)** and 183 dpe **(C)**. Parasite stages observed at 29 dpe included luminal proliferative trophozoites (A_1_), disporoblasts with mature spores (A_2_), and intratissue proliferative trophozoites (A_3_). Note the high abundance of intraepithelial lymphocytes in A_1_ and A_2_. Parasite stages observed at 57 dpe included proliferative trophozoites, both luminal (B_1_) and intraepithelial (B_2_). Parasite stages observed at 183 dpe were exclusively luminal proliferative trophozoites (C_1_, C_2_), with severe intraepithelial lymphocyte infiltration (C_3_). In ALLO fish, submucosal hyperplasia was highest at 57 dpe **(E)**. Abundant luminal parasite stages were observed at 29 dpe, including both proliferative trophozoites (D_1_) and disporoblasts with mature spores (D_3_). Intraepithelial proliferative trophozoites (D_2_) were also present. At 57 dpe, intraepithelial proliferative trophozoites and lymphocytes (E_1_), and luminal proliferative trophozoites and disporoblasts with mature spores (E_2_) were noted. At 183 dpe, intraepithelial and luminal proliferative trophozoites (F_1_), severe high intraepithelial lymphocyte infiltration (F_2_), and luminal disporoblasts with mature spores (F_3_) were found. Black arrowheads = luminal proliferative trophozoites; white arrowheads = luminal disporoblasts with mature spores; black arrows = intratissue proliferative trophozoites; asterisks = intraepithelial lymphocyte infiltration. Giemsa stained sections. Scale bars in **(A–C)** and **(D–F)** = 50 µm; scale bars in A_1_–A_3_, B_1_–B_2_, C_1_–C_3_, D_1_–D_3_, E_1_–E_2_, and F_1_–F_3_ = 20 µm.

Infection was detected in the gills of exposed fish by qPCR as early as 1 dpe, in both fish strains. When intestines were first sampled at 8 days post-exposure, only one ALLO sample was positive; by 15 dpe, all exposed fish were PCR positive. Fish remained infected throughout the 183 days of the experiment. In the gill sections of 1 dpe and 8 dpe fish, no *C. shasta* stages could be found. In intestines, however, proliferative parasite stages were found from 15 dpe onward (**Figures 1**, **2**; **Supplementary Table 2**). Low numbers of proliferative trophozoites migrating through the lamina propria–submucosa or epithelium were observed in SYM fish from 15 dpe to 57 dpe, whereas in ALLO fish, they were present from 15 dpe to 183 dpe (**Figures 1A**, **2**). Two different developmental stages of *C. shasta*, namely, proliferative trophozoites and disporoblasts with mature spores, were observed in the intestinal lumen of exposed fish, either free or attached to the mucosal surface (**Figures 1B**, **2**). Infection intensity with such luminal trophozoites was highest in SYM fish at 29 dpe and decreased later on. In ALLO fish, highest counts of luminal trophozoites occurred also at 29 dpe and were sustained until 183 dpe, a time point at which *C. shasta* trophozoites were significantly more numerous in ALLO than in SYM. Parasite disporoblasts appeared first at 29 dpe in both trout strains, though with significantly higher numbers in ALLO than in SYM. After 29 dpe, *C. shasta* disporoblasts persisted only in ALLO fish (**Figures 1B**, **2**). Limited mortality occurred following exposure in both exposed SYM and ALLO groups but appeared to be non-specific and not related to *C. shasta* infection. All control fish were negative for *C. shasta*.

### 3.2 Histopathological observations

**Figure 3:**
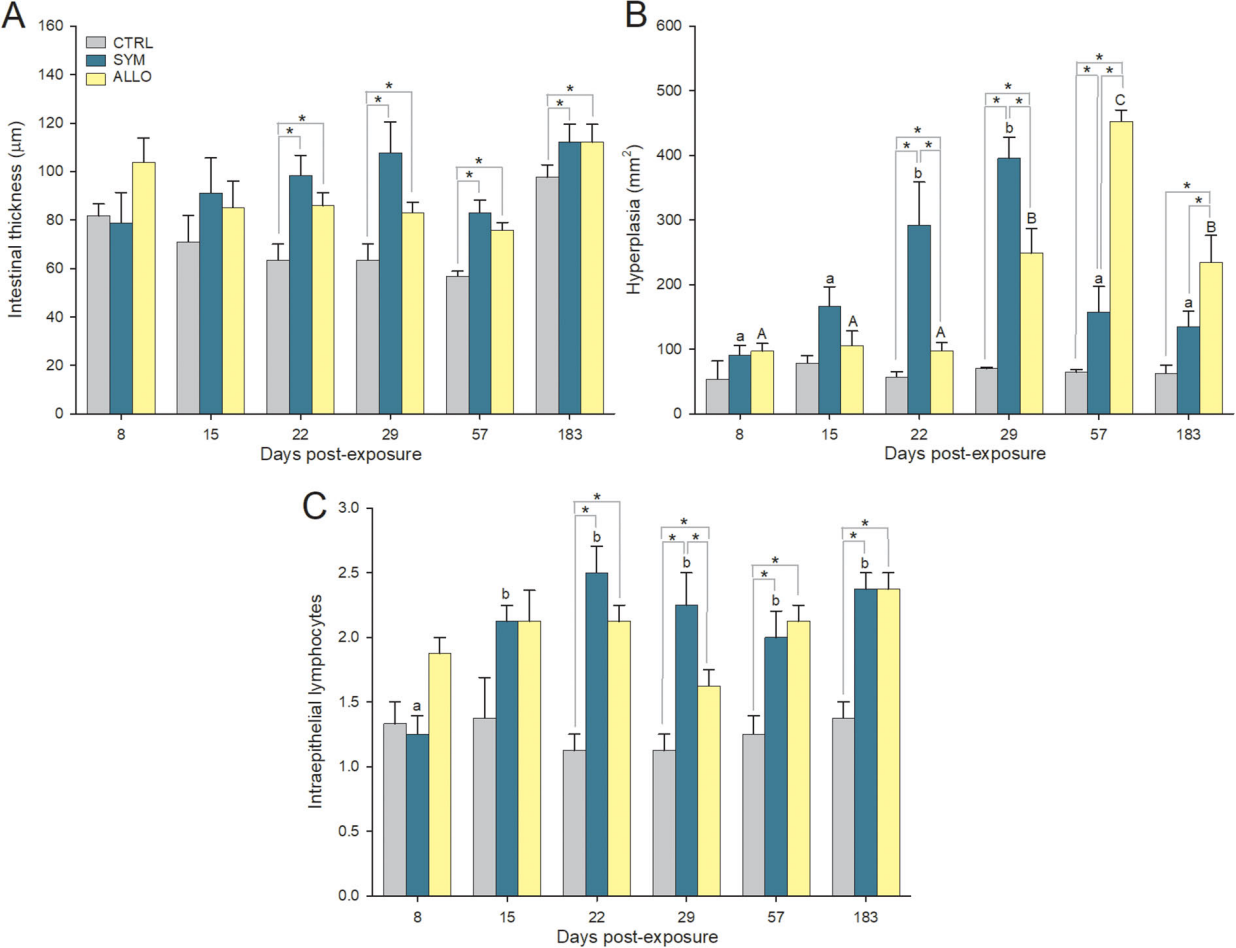
Intestinal histopathological alterations in control unexposed (CTRL) and *Ceratonova shasta*-exposed sympatric (SYM) and allopatric (ALLO) steelhead trout. **(A)** Intestinal thickness including *muscularis* layers*, stratum granulosum*, *stratum compactum*, and lamina propria–submucosa. **(B)** Hyperplasia of the lamina propria–submucosa. **(C)** Lymphocyte epithelial infiltration scored from 0 (absence) to 3 (very abundant, meaning 25–30 intraepithelial lymphocytes per microscope field at 500× magnification). Different lowercase and uppercase letters stand for significant differences among sampling times within SYM or ALLO fish, respectively. Asterisks stand for significant differences between two experimental groups within a time post-exposure. *P* < 0.05.

In gills, no significant histopathological alterations were observed. In intestines, an overall increase in thickness was observed from 22 dpe onward in exposed steelhead trout due significant hyperplasia of the lamina propria–submucosa (**Figures 3A, B**). Thickness of the underlying intestinal layers (*muscularis*, *stratum granulosum*, and *stratum compactum*) did not change (**Supplementary Figure 1**). Hyperplasia was significant in SYM fish earlier than in ALLO, in which it became significant only from 29 dpe onward. By 57 dpe, intestinal hyperplasia peaked in ALLO fish, but had subsided in SYM fish (**Figures 2**, **3B**). Overall, the increased intestinal thickness due to the hyperplasia of the lamina propria–submucosa was significant in both strains, regardless of the infection timing, when compared to control unexposed (CTRL) fish (**Supplementary Figures 2A, B**). The inflammatory response in the intestines of exposed fish included a significant infiltration of lymphocytes in the intestinal epithelium (**Figures 2**, **3C**; **Supplementary Figure 2C**). This increase of intraepithelial lymphocytes (IELs) occurred in both fish strains and was significant in SYM fish from 15 dpe on and in ALLO fish from 22 dpe onward. Sections of CTRL intestines are shown in **Supplementary Figure 3** for comparison.

### 3.3 Lymphocyte quantification

**Figure 4:**
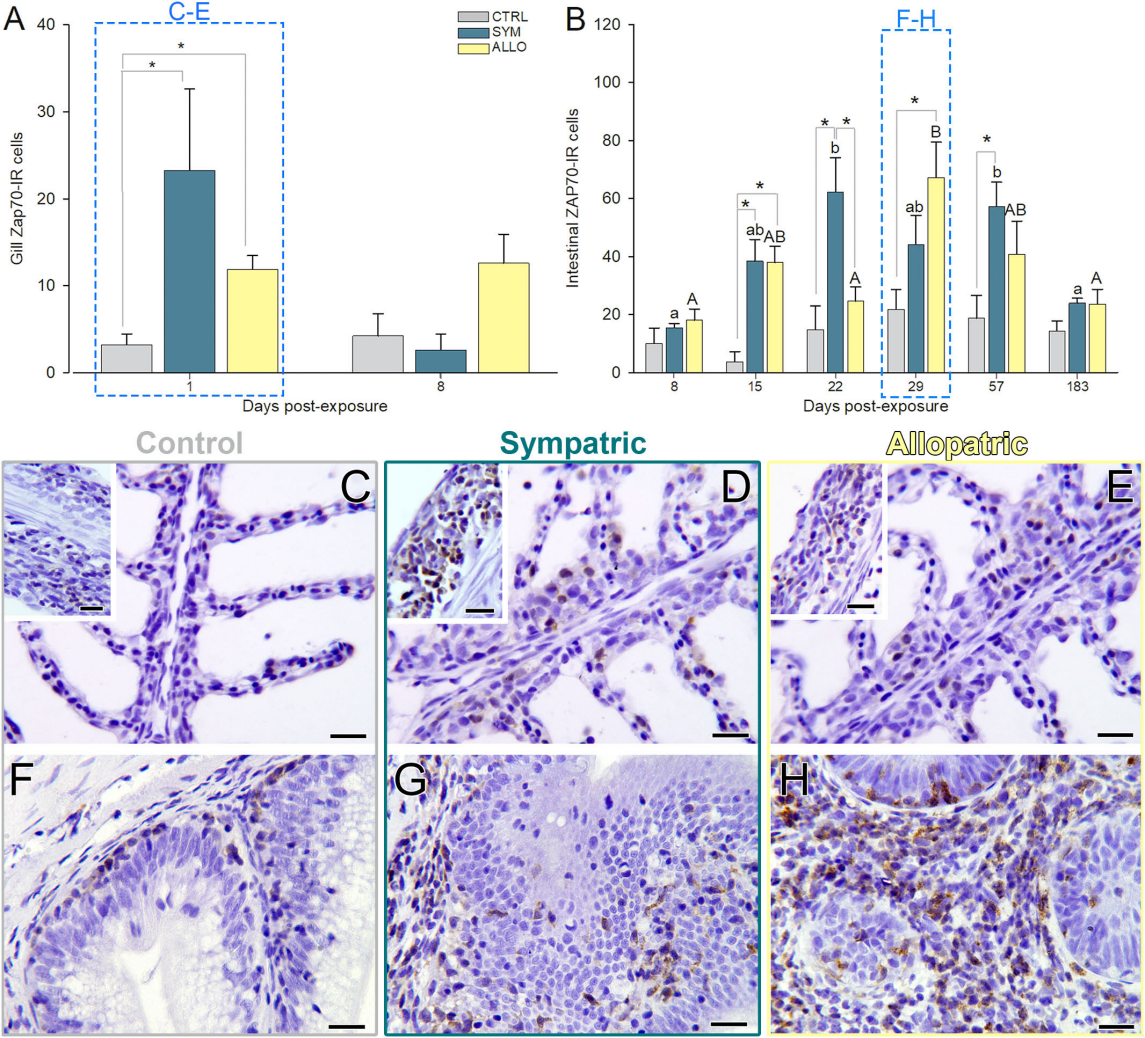
Zap70 immunoreactive T cells (Zap70-IR; brown stain) in intestines **(A, C–E)** and gills **(B, F–H)** of control unexposed (CTRL; **C, F**) and *Ceratonova shasta* exposed sympatric (SYM; **D, G**) and allopatric (ALLO; **E, H**) steelhead trout. **(A, B)** Different lowercase and uppercase letters stand for significant differences among sampling times within SYM or ALLO fish, respectively. Asterisks stand for significant differences between two experimental groups within a time post-exposure (*P* < 0.05). Microphotographs of sampling times highlighted in dashed boxes are represented in **(C–H)**: Intestinal sections correspond to 29 days post-exposure (dpe) and gill sections correspond to 1 dpe; each insert shows an area of the non-lamellar filament surface. Hematoxylin stained sections. Scale bars = 20 µm.

Different lymphocyte types were labeled and quantified in the gill and intestinal tissues of the experimental steelhead trout (summarized in **Supplementary Figures 2D–F**, **4**). Numbers of Zap70 immunoreactive T cells were significantly higher in the gills of exposed SYM and ALLO fish at 1 dpe (**Figure 4A**). These T cells were found spread in the gill filament and lamellae (**Figures 4C–E**). In the intestines of both SYM and ALLO exposed fish, T cell numbers were significantly higher than in control fish at 15 dpe (**Figure 4B**; **Supplementary Figure 2D**). T cell abundance peaked in exposed SYM intestines at 22 dpe, whereas in exposed ALLO, it peaked later at 29 dpe (**Figures 4F–H**). By 183 dpe, intestinal numbers of T cells in exposed fish were restored to the levels of control fish in both fish strains. In control intestines, constitutive numbers of T cells remained unchanged, and T cells were found mostly restricted to the base of the epithelium, whereas in exposed animals, they appeared invading the lamina propria–submucosa and infiltrated in the epithelium.

**Figure 5:**
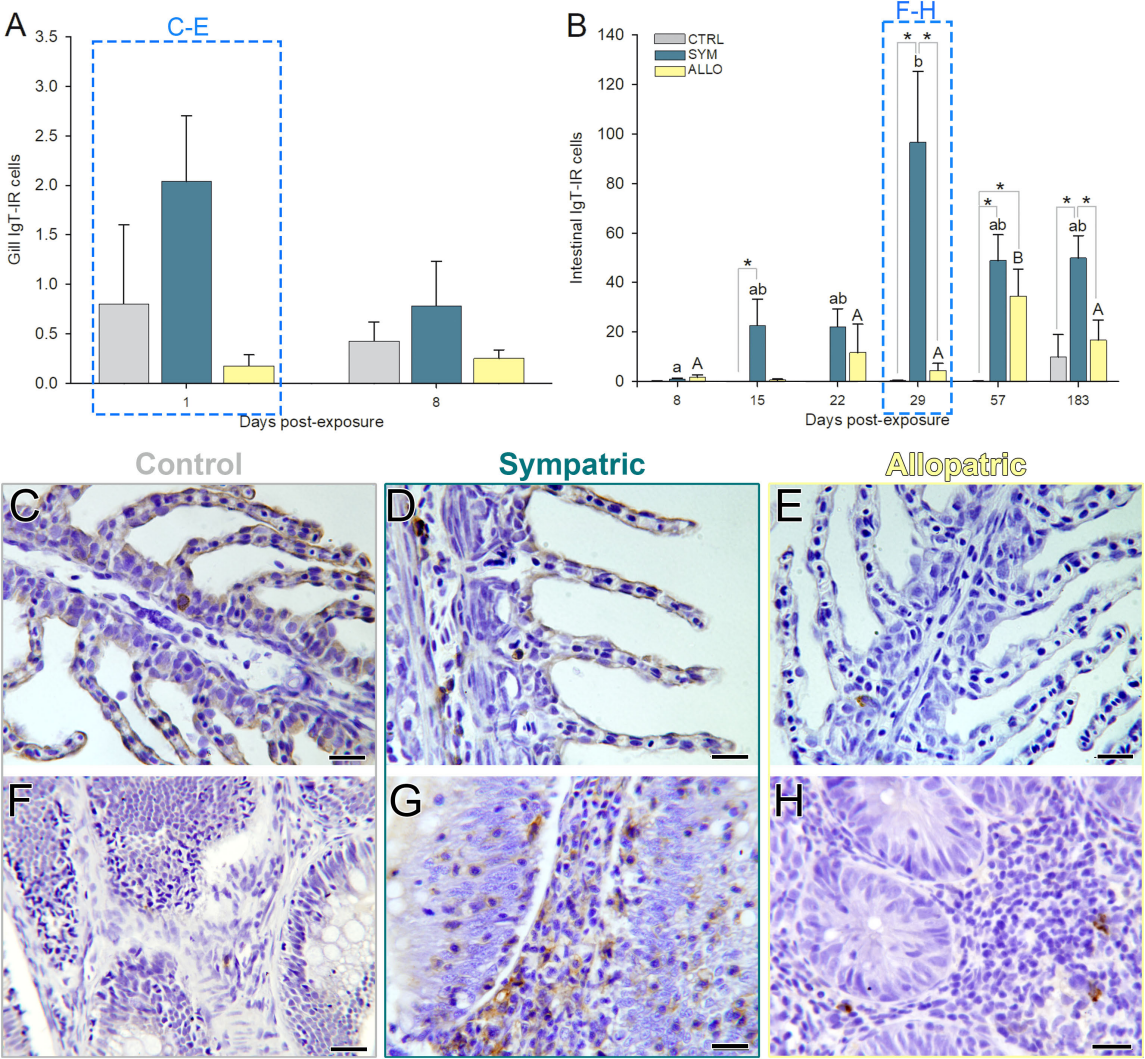
IgT immunoreactive B cells (IgT-IR; brown stain) in intestines **(A, C–E)** and gills **(B, F–H)** of control unexposed (CTRL; **C, F**) and *Ceratonova shasta* exposed sympatric (SYM; **D, G**) and allopatric (ALLO; **E, H**) steelhead trout. **(A, B)** Lowercase and uppercase letters indicate significant differences among sampling times within SYM or ALLO fish, respectively. Asterisks indicate significant differences between two experimental groups within a time post-exposure (*P* < 0.05). Microphotographs of sampling times highlighted in dashed boxes are represented in **(C–H)**: Intestinal sections correspond to 29 days post-exposure (dpe) and gill sections correspond to 1 dpe. Hematoxylin stained sections. Scale bars = 20 µm.

IgT immunoreactive B cells in the gills were scarce, and their numbers did not change significantly over time (**Figure 5A**) but were significantly higher in SYM than in ALLO upon *C. shasta* exposure (**Supplementary Figure 4B**). Interestingly, the IgT immunolabel was noted at the surface of gill lamellae of control and exposed SYM fish but was absent in ALLO gills (**Figures 5C–E**). In intestines, a consistent increase of IgT^+^ B cells was observed in exposed SYM fish from 29 dpe onward and in exposed ALLO only at 57 dpe (**Figure 5B**). Only few dispersed IgT^+^ B cells were observed in the intestines of control and exposed ALLO fish, while in exposed SYM intestines, IgT^+^ B cell aggregates were observed in the lamina propria–submucosa and abundant IgT^+^ B cells infiltrated the epithelial layer (**Figures 5F–H**). Overall, SYM fish presented the highest IgT^+^ B cell numbers in intestines, and in ALLO fish, a less intense but still significant increase of IgT^+^ B cells was observed (**Supplementary Figure 2E**).

**Figure 6:**
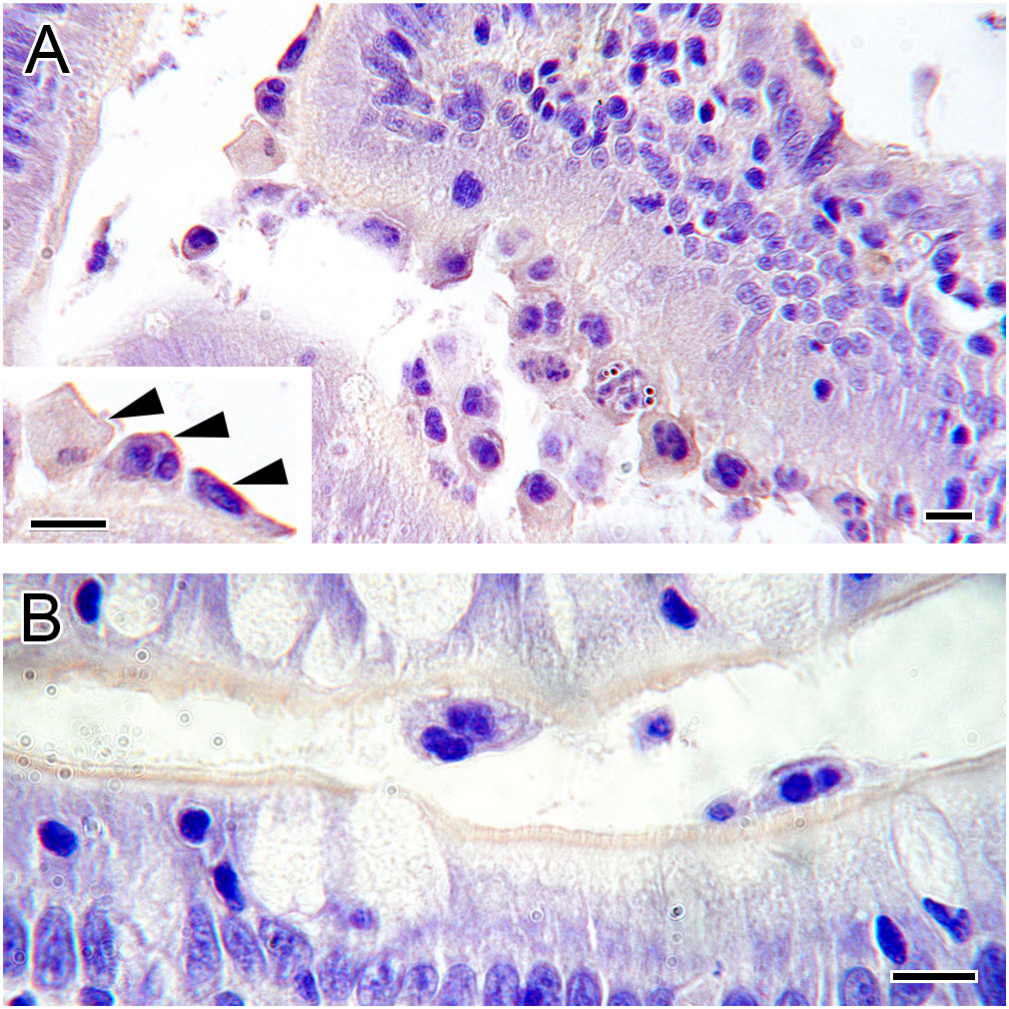
Ig immunoreactivity on intestinal *Ceratonova shasta* stages. **(A)** IgT immunolabeling observed on luminal parasite stages in a sympatric steelhead host. Note the surface labeling on parasite trophozoites attached to the intestinal mucosa (arrowheads). **(B)** Absence of IgD immunolabeling on parasite stages in a sympatric steelhead host. Note the light positive label on the intestinal brush border. Scale bars = 10 µm.

In addition to B cell labeling, IgT immunolabeling was also observed on the surface of luminal intestinal parasite stages, indicating that those parasite stages were IgT-coated (**Figure 6A**). This label was, however, inconsistent among the different parasite stages within the same intestinal sample and among SYM and ALLO fish.

**Figure 7:**
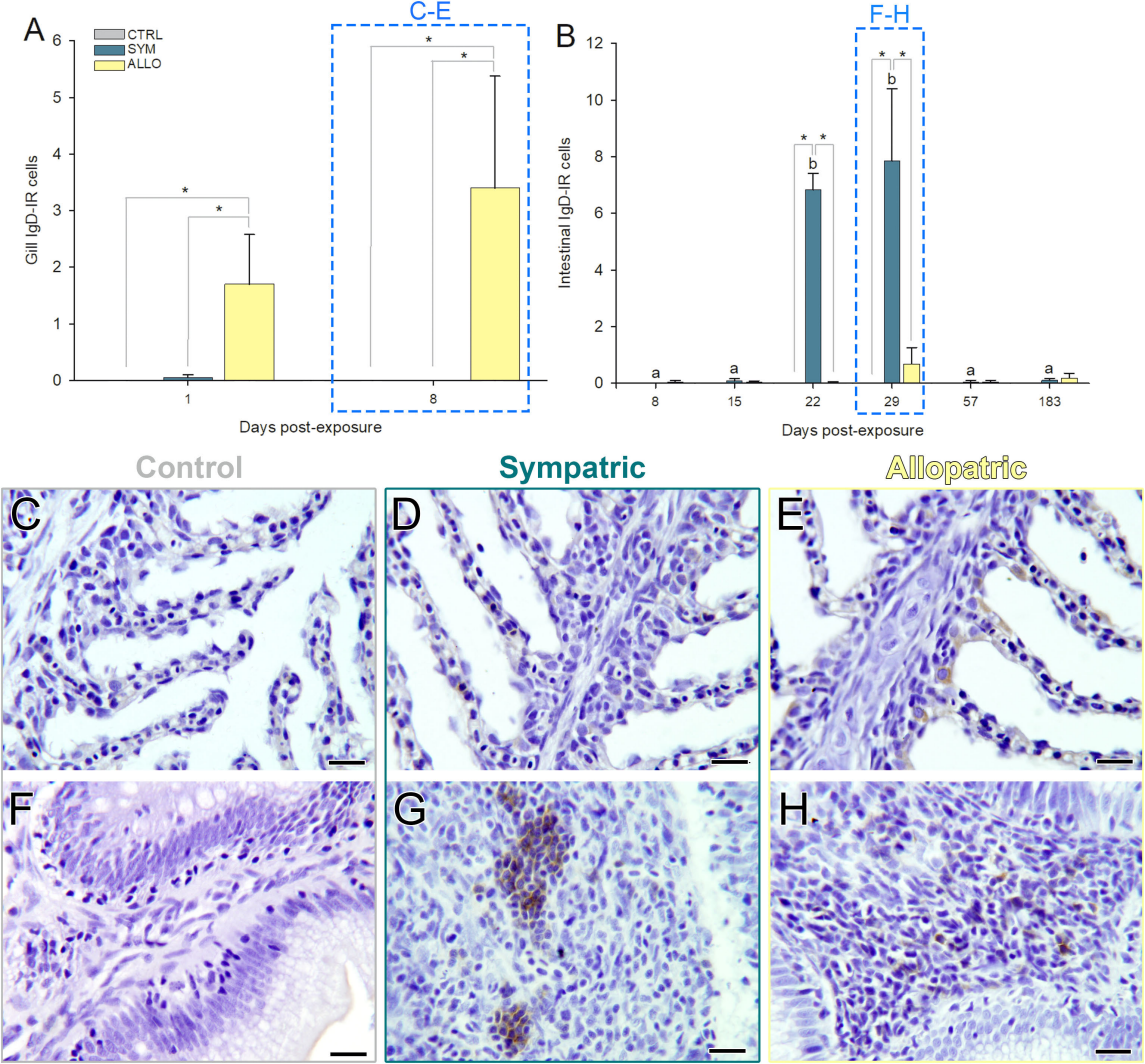
IgD immunoreactive B cells (IgD-IR; brown stain) in intestines **(A, C–E)** and gills **(B, F–H)** of control unexposed (CTRL; **C, F**) and *Ceratonova shasta* exposed sympatric (SYM; **D, G**) and allopatric (ALLO; **E, H**) steelhead trout. **(A, B)** Lowercase letters indicate significant differences among sampling times within SYM fish. Asterisks indicate significant differences between two experimental groups within a time post-exposure (*P* < 0.05). Microphotographs at sampling times highlighted in dashed boxes are represented in **(C–H)**: Intestinal sections correspond to 29 days post-exposure (dpe) and gill sections correspond to 8 dpe. Hematoxylin stained sections. Scale bars = 20 µm.

IgD immunoreactive B cells were the scarcest lymphocyte type in both tissues. However, IgD^+^ B cell numbers in gills were significantly higher in exposed ALLO fish than in the other experimental groups (**Figures 7A, C–E**; **Supplementary Figure 4C**). At the intestinal level, higher numbers of IgD^+^ B cells were registered only in exposed SYM fish at 22 and 29 dpe (**Figure 7B**; **Supplementary Figure 2F**), and these formed cell aggregates in the lamina propria–submucosa (**Figures 7F–H**). No IgD immunolabeling was observed on parasite stages (**Figure 6B**).

### 3.4 Specific circulating antibodies

**Figure 8:**
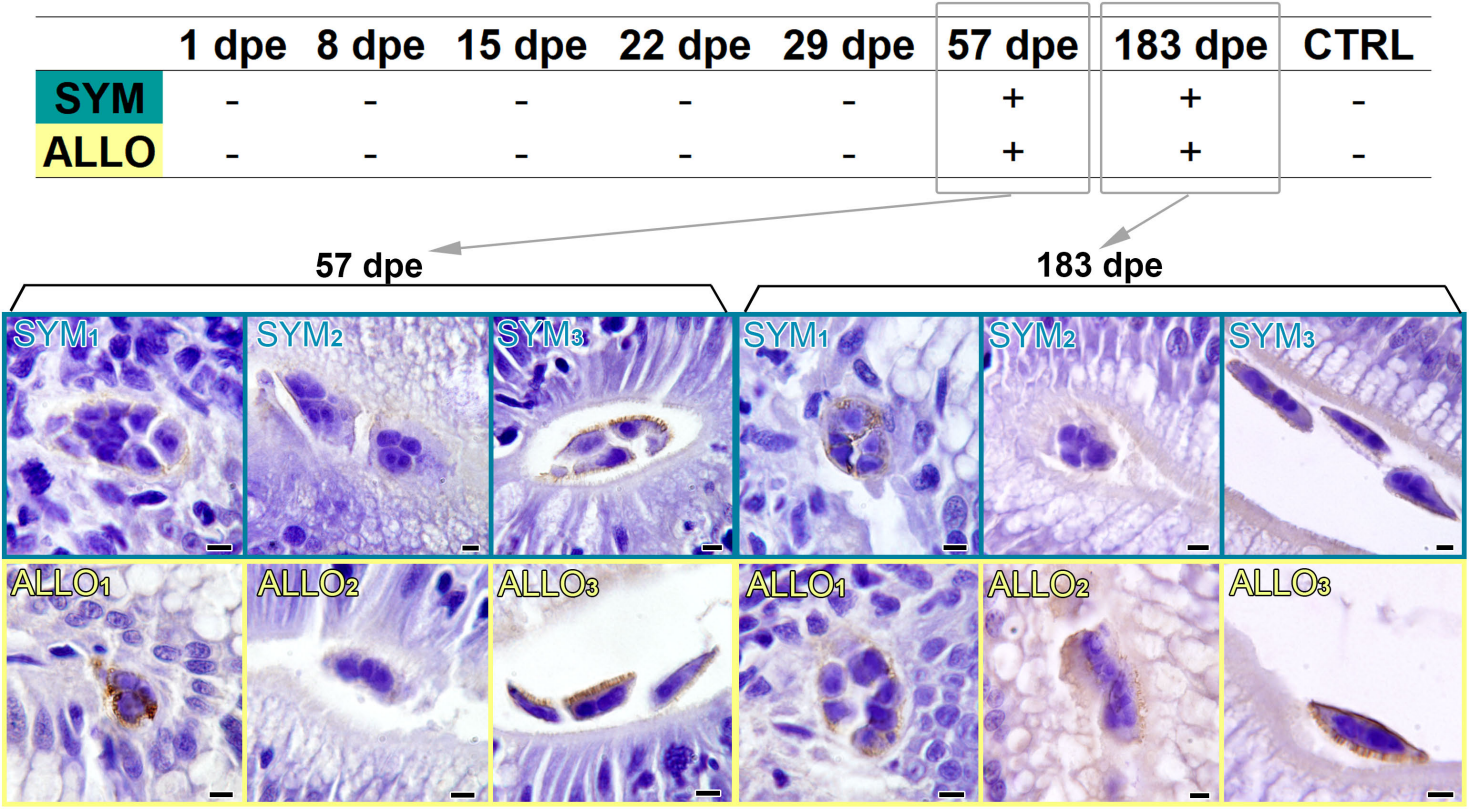
*Ceratonova shasta* immunolabeling with specific serum IgM of steelhead trout exposed to parasite at different days post exposure (dpe) and of control unexposed fish (CTRL). Serum pools of four different animals were used for each immunoreaction. Parasite stages immunoreacted only with serum pools in 57 and 183 dpe samples. Note the different intensity and location of parasite labeling in intratissue trophozoites (SYM_1_, ALLO_1_), luminal trophozoites attached to the mucosal surface (SYM_2_, ALLO_2_), and free luminal trophozoites (SYM_3_, ALLO_3_). Scale bars = 5 µm.

Parasite labeling with specific IgT of the tested serum pools was negative for all times and both fish strains. By contrast, parasite stages were specifically labeled with serum IgM of *C. shasta*-exposed SYM and ALLO fish at 57 and 183 dpe (**Figure 8**). Differences in parasite immunoreactivity were noted depending on the stage and its location. Only proliferative trophozoites were detected by the circulating IgM (not disporoblasts), and in general, luminal trophozoites attached to the mucosal surface of the intestine were the less immunoreactive ones, compared to free luminal trophozoites and to migrating intratissue trophozoites. Immunolabel was apparently located on the surface of *C. shasta* and in the cytoplasm of secondary daughter cells. Parasite pseudopodia were intensely labeled, especially in the luminal trophozoites. Label intensity was more intense with SYM sera than with ALLO sera only for some parasite stages.

### 3.5 Ultrastructural observations

**Figure 9:**
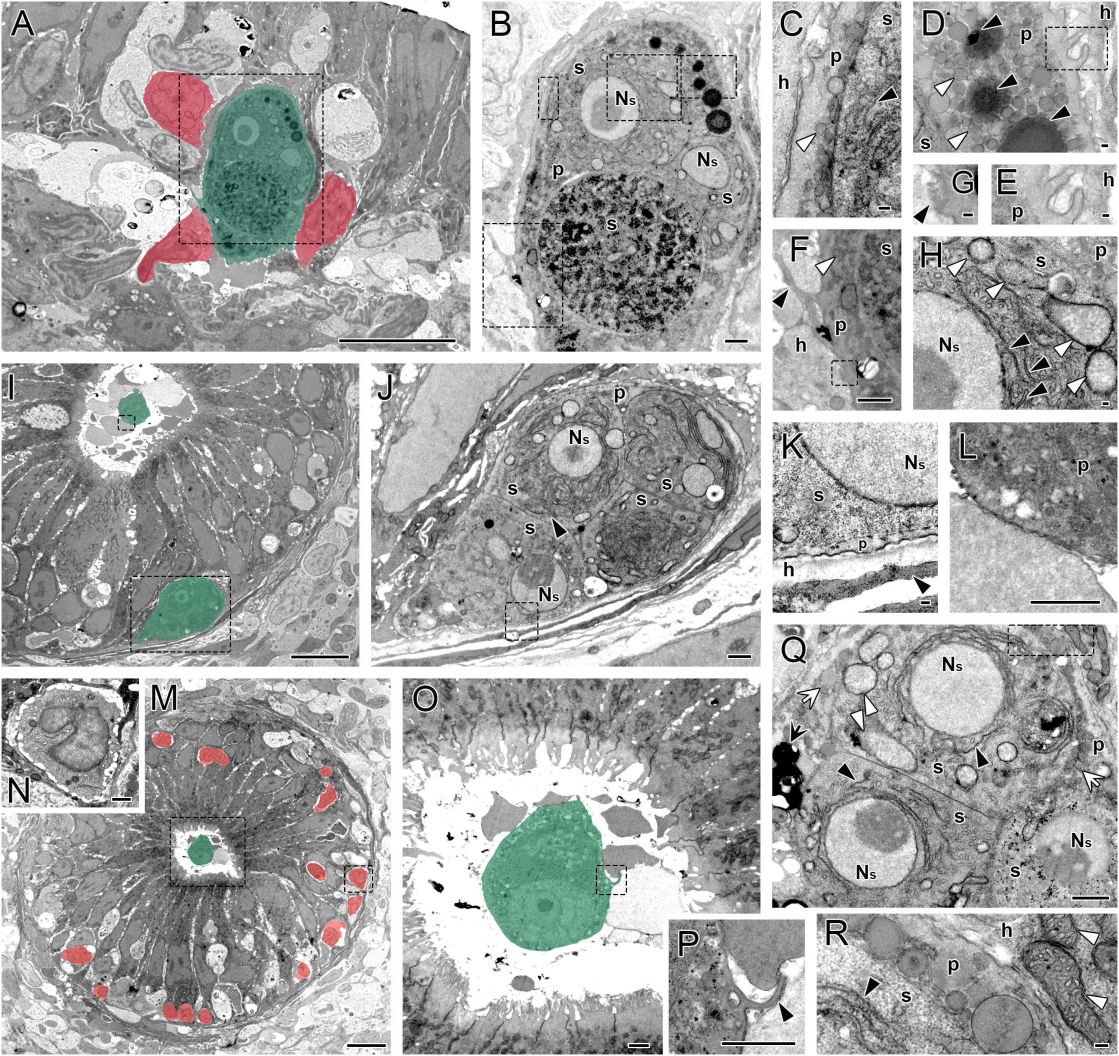
*Ceratonova shasta* proliferative trophozoites in allopatric *Oncorhynchus mykiss* intestines at 3–4 months post exposure. In lower magnification images, parasite stages are colored green and fish lymphocytes are colored red. **(A)** *C*. *shasta* trophozoite located in the intestinal epithelium, in close contact with fish lymphocytes. **(B)** Higher magnification of the parasite in **(A)** showing three secondary (s) cells within the primary (p) cell. **(C)** Higher magnification of the area in the upper left dashed square in **(B)** showing the contact between host cell and p cell with abundant cytoplasmic vesicles (white arrowhead). Note the ribosome-packed cytoplasm and abundant rough endoplasmic reticulum (RER, black arrowhead) in the s cell. **(D)** Higher magnification of the area in the upper right dashed square in **(B)** showing the p cell cytoplasm with abundant cytoplasmic vesicles (white arrowheads) and electrodense lipid droplets (black arrowheads). Note the p cell cytoplasmic protrusions in the dashed square, which is magnified in **(E)**. **(F)** Higher magnification of the area in the lower dashed square in **(B)** showing the contact area between p parasite cell and host lymphocyte. Note the p cell with pseudopod (black arrowhead) reaching between host cells and abundant cytoplasmic vesicles (white arrowhead). Within the p cell, the s cell harbors abundant cytoplasmic ribosomes. The dashed square is magnified in **(G)**, showing the irregular contact surface between the p cell and a lymphocyte, with numerous ridges and blebbings (black arrowhead). **(H)** Higher magnification of the area in the middle-dashed square in **(B)** showing the ribosome-packed cytoplasm and abundant RER (black arrowhead) and mitochondria (white arrowheads) in the s cell. **(I)** Intestinal cross-section with a *C*. *shasta* luminal stage and an intraepithelial stage close to the basal membrane. **(J)** Higher magnification of the lower dashed square in **(I)** showing a trophozoite with four internal s cells. **(K)** Higher magnification of the dashed square in **(J)** showing detail of the junction between trophozoite and epithelial basal membrane (black arrowhead) of the host intestine. **(L)** Higher magnification of the upper dashed square in **(I)** showing detail of the luminal stage membrane in contact with luminal host cell debris. **(M)** Intestinal cross-section with luminal trophozoite and abundant infiltrated intraepithelial lymphocytes. **(N)** Higher magnification of the right dashed square in **(M)** showing detail of an intraepithelial lymphocyte. **(O)** Higher magnification of the left dashed square in **(M)** showing trophozoite in the intestinal lumen. Note the dashed square magnified in **(P)** showing parasite’s pseudopod contacting luminal host cell debris (black arrowhead). **(Q)** Intraepithelial trophozoite consisting of three s cells within the p cell. Note the abundant RER (black arrowhead) and the mitochondria (white arrowheads) in s cells, and the cytoplasmic lipid inclusions (black arrow) and vesicles (white arrows) in the p cell. **(R)** Higher magnification of the dashed square in **(Q)** showing details of RER (black arrowhead) in the ribosome-packed cytoplasm of the s cell and mitochondria (white arrowheads) in the neighboring host cell. p, primary cell; h, host cell; s, secondary cell; N_S_, nucleus of the s cell. Scale bars in **(A, I, M)** = 10 µm; in **(B, F, J, L, N–Q)** = 1 µm; in **(C–E, G, H, K, R)** = 100 nm.

Only proliferative trophozoites of *C. shasta* were detected in the intestinal samples analyzed by TEM. These stages consisted of a primary cell encompassing up to four secondary daughter cells (**Figure 9**). Intraepithelial infiltration of lymphocytes was evident in the intestines, and trout lymphocytes were observed surrounding parasite stages, in direct contact with their primary cell (**Figures 9A–D**). The parasites’ primary cells presented pseudopodia and cytoplasmic protrusions extending toward or in between host cells (**Figures 9C, H, O**), and the primary cell membrane contacting lymphocytes or other host cells presented numerous ridges and blebbing (**Figures 9C, F, G**). The primary cell cytoplasm contained lipid inclusions and numerous vesicles, whereas the cytoplasm of developing secondary cells was densely packed with ribosomes, abundant rough endoplasmic reticulum (RER), and mitochondria (**Figures 9C, F, G, I, M, S**). The nuclei of the secondary cells showed a large eccentric nucleolus (**Figures 9E, L, R**). Free parasite stages were also observed in the intestinal lumen surrounded by undifferentiated cell debris (**Figures 9J, N, P**), and some also presented extended pseudopodia (**Figure 9O**).

## 4 Discussion

The genotype 0 lineage of *Ceratonova shasta* has the ability to proliferate and transmit without causing the death of its fish host (Atkinson and Bartholomew, 2010a; Alama-Bermejo et al., 2019; Taggart-Murphy et al., 2021) and thus offers a model to investigate parasite tolerance through the factors involved in both myxozoan virulence and host resistance. **Graphical Abstract** summarizes the main findings of this study, in which we investigated the host–parasite relationship from the perspective of the host. In the current infection challenge, *C. shasta* G0 did not cause mortality in either ALLO or SYM hosts. In the gills, although *C. shasta* G0 was not observed by histology and no histopathological signs were observed, PCR+ fish were detected from both SYM and ALLO steelhead groups at 1 dpe, consistent with previous observations that the gills are the sites of initial parasite invasion (Bjork and Bartholomew, 2010). Earlier studies (Bjork and Bartholomew, 2010; Bjork et al., 2014) concluded that resistance to *C. shasta* GI invasion did not occur in the gills of either SYM or ALLO chinook salmon, and parasite morphology, replication, and migration from the gills into the intestine appeared similar between susceptible ALLO rainbow trout and resistant SYM chinook salmon.

### 4.1 Intestinal parasite proliferation and sporogenesis are restricted in the sympatric fish

In the intestine, both fish strains were PCR+ from 15 dpe onward throughout the entire study. Differences in infection dynamics were observed between strains, and these differences influenced the period when sporulation occurred. In SYM steelhead trout, trophozoite proliferation was lower and sporogenesis seemed occasional, compared to the ALLO steelhead. These observations may suggest that *C. shasta* G0 has adjusted its life cycle in its SYM host through their long co-evolutionary history, that the SYM host has developed a differential, perhaps more effective immune response against the parasite, or that both of these occur and complement each other. Early sporogenesis in resistant anadromous hosts supports completion of the parasite’s life cycle while hosts are still in the river ecosystem and spores can thus encounter the annelid host. Nevertheless, proliferative trophozoites persisting in the intestinal lumen of SYM fish might provide an opportunity for the parasite to remain dormant until some triggering occurs for sporulation. This strategy also entails parasite adaptation to a coelozoic lifestyle, which is generally less pathogenic for the host. The long host–parasite co-evolution of genotype 0 has been assumed from its high specificity for the putative ancestral hosts, low virulence, and phylogenetic reconstruction and genetic structure models (Breyta et al., 2020; Bartholomew et al., 2022). Under these circumstances, the introduction of ALLO fish stocks in water bodies endemic for *C. shasta* G0 might have provided a new host scenario in which parasite proliferation and sporogenesis might be unleashed. Transcriptomic, proteomic, and epitope analysis may help to explain the underlying mechanisms for the differential expression of parasite virulence factors and life stage-specific markers in SYM and ALLO steelhead.

There is no previous data on the differential infection dynamics of *C. shasta* G0 in SYM and ALLO fish hosts, although studies with genotypes I and II have provided interesting results. Upon exposure to a lower actinospore dose of the more virulent *C. shasta* GIIR, resistant SYM steelhead effectively contained parasite proliferation at 7 dpe, whereas in susceptible ALLO steelhead, parasite replication was still exponentially increasing at 21 dpe (Barrett and Bartholomew, 2021). In those fish, *C. shasta* GIIR stages invaded the intestinal mucosa and submucosa, as was also reported for *C. shasta* GI in SYM and ALLO chinook salmon (Bjork and Bartholomew, 2010). In contrast, in this study, proliferation of *C. shasta* G0 continued apparently unrestricted through the 183 days of the study only in the intestinal lumen of SYM and ALLO steelhead, and sporogenesis was limited to the early infection (29 dpe) only in SYM hosts whereas it continued in ALLO until the end of the experiment. A previous study evidenced that *C. shasta* G0 differed from the other genotypes in having a low proliferation rate, lower spore production, and less active parasite stages (Alama-Bermejo et al., 2019). Cell movement of motile myxozoan stages has been attributed to tissue invasion and immune evasion strategies (EL–Matbouli et al., 1995; Alama-Bermejo et al., 2012; Hartigan et al., 2016). Indeed, the virulent genotypes (I and II) which proliferate in the lamina propria–submucosa and epithelium rely on their high motility to evade host immune factors of the gut-associated immune tissue (GALT), as completion of their life cycle depends on rapid proliferation and spore release, before host death (Alama-Bermejo et al., 2019, 2020). However, high motility may not be crucial for the success of *C. shasta* G0 infections, which differ from the other genotypes in their high luminal prevalence and low tissue invasion.

### 4.2 Intestinal hyperplasia is a sign of inflammatory dysregulation in allopatric fish

The intestinal histopathology of ALLO steelhead trout upon *C. shasta* G0 infection involved mild submucosal inflammation with moderate infiltration of immune cells and minimal epithelial damage (Taggart-Murphy et al., 2021). In the present study, two main characteristic inflammatory signs were observed in the intestine upon exposure to *C. shasta* G0, regardless of the fish strain: submucosal hyperplasia and intraepithelial infiltration of lymphocytes. The submucosal hyperplasia overlapped with the presence of proliferative trophozoites migrating through the intestinal layers in both host strains. Thus, hyperplasia in SYM fish seemed mostly resolved from 29 dpe onward. In ALLO fish, however, the onset of the hyperplasia was delayed, and the condition persisted until 183 dpe, pointing to an inflammatory dysregulation when compared to SYM. Previous studies have also attributed extensive intestinal inflammation and lymphocytic infiltration to the proliferation of *C. shasta* GIIR stages within the tissue accompanied by an upregulation of proinflammatory cytokine expression (Taggart-Murphy et al., 2021). However, epithelial disruption and total breakdown of the intestinal barrier, as reported during *C. shasta* GIIR infection in ALLO steelhead trout (Barrett and Bartholomew, 2021), did not occur in either ALLO or SYM hosts upon exposure to *C. shasta* G0.

The inflammatory response protects the fish host by inducing repair mechanisms through recruitment of inflammatory filtrates and proliferation of local immune effectors (Soliman and Barreda, 2023). However, inappropriate regulation may result in an excessive inflammatory response and pathogenesis as reported from some myxozoan infections (Sitjà-Bobadilla et al., 2015; Holzer et al., 2021). Fine tuning of the regulatory response during host–parasite co-evolution may be responsible for the histopathological differences observed in the intestines of ALLO and SYM steelhead during *C. shasta* G0 infection. This immune regulation/dysregulation is particularly evident in two intestinal myxozoan genera that have been the focus of research due to their devastating impact on aquaculture fish species, *Ceratonova* and *Enteromyxum*. The adaptive immune response of gilthead seabream (*Sparus aurata*) resistant to reinfection with *Enteromyxum leei* displayed a post-inflammatory gene expression profile in the intestine involving downregulation of *il1β* and *hsp90α* and upregulation of *il10* (Picard-Sánchez et al., 2019). This might hint toward an early scenario of inflammation resolution occurring only during *C. shasta* G0 infections, in which the inflammatory response elicited by any parasite stages migrating through the tissue is effectively suppressed or regulated by the fish hosts. Thus, a stronger and earlier post-inflammatory profile would be expected in SYM intestines than in ALLO. Once more, further studies on the differential transcriptomics of SYM and ALLO steelhead are needed to identify subtle differences in their regulatory response at the intestinal level, with special focus on regulatory T cell markers and anti-inflammatory cytokines, as well as on innate factors.

### 4.3 Intestinal T cell response is triggered earlier in the sympatric fish

T cell activation is an early event in the adaptive immune response, occurring upon specific antigen recognition through the T cell receptor, which encompasses recruitment and activation of the Zap70 kinase. Zap70 kinase is considered a *pan* T cell marker, expressed on CD8^+^ cytotoxic T cells (CTLs) as well as on CD4^+^ helper T cells, mediators of B cell activation and antibody production through cytokine release (Laing and Hansen, 2011). Intestinal T cell recruitment/proliferation was evidenced in the exposed fish by Zap70 immunolabeling. An increase in T cells was detected in both host strains when trophozoites were first detected in the intestinal tissue. Zap70^+^ T cells were mostly consistent with intraepithelial T lymphocytes in their morphology and location, but we cannot discount the presence of CTLs, capable of directly killing parasite cells. For now, we have demonstrated a T cell response against *C. shasta* G0, but we cannot discern to what extent the later T cell response in ALLO hosts contributes to slower parasite clearing in the intestinal tissue and to the subsequent unimpeded luminal parasite proliferation beginning at 29 dpe. The analysis of transcriptional profiles of T cell subsets will aid in understanding the T cell response that takes place in the intestines of SYM vs. ALLO steelhead trout after *C. shasta* G0 exposure, as previously reported for CTL signature gene expression in *C. shasta* GIIR infection (Barrett and Bartholomew, 2021) and in resistant gilthead seabream to *E. leei* reinfection (Picard-Sánchez et al., 2019).

The available literature on T cell responses to fish myxozoans is limited. However, activation of the IFNγ-signaling pathway mediated by T_H1_ cells seems a common response in resistance against myxozoan infections. An effective T_H1_ response was assumed from the ALLO rainbow trout resistant to *C. shasta* G0 infection due to the intestinal *ifnγ* upregulation (Taggart-Murphy et al., 2021) and steelhead trout resistant to the virulent *C. shasta* GIIR (Barrett and Bartholomew, 2021). Thus, early specific *C. shasta* recognition might be an essential aspect for immune resistance. The studied enteromyxosis models also provide a range of pathogenesis and immune responses in naïve vs. resistant gilthead seabream and turbot (*Scophthalmus maximus*) against *E. leei* and *Enteromyxum scophthalmi*, respectively. In the highly susceptible naïve turbot, exhaustion and downregulation of T_H1_, T_H17_, and CTL gene markers (Robledo et al., 2014) follow the initial T_H1_ response (Ronza et al., 2016), whereas in naïve gilthead seabream with a less severe disease course, the T cell response encompasses a strong intestinal recruitment of Zap70^+^ and Tbet^+^ T lymphocytes along with expression profiles of CTL, T_H1_, and T_H2_ responses (Piazzon et al., 2018). Furthermore, gilthead seabream resistance against enteromyxosis was associated with mucosal recruitment of T cells from the head kidney (Picard-Sánchez et al., 2019). Also, gibel carp (*Carassius auratus gibelio*) with a severe *Myxobolus hunghuensis* pharynx infection, displayed T cell recruitment into the infection site along with T_H1_ and T_H2_ transcriptional profiles (Zhao et al., 2019). Thus, among myxozoonoses, an early activation of the T cell response in mucosal infection sites is supported systemically, and its correct regulation seems critical for myxozoan resistance and disease development. A remarkable example for effective T cell activation is resistant Atlantic salmon (*Salmo salar*) re-exposed to the muscle-infecting *Kudoa thyrsites*, in which recruitment of CD8^+^ CTLs to the infection site aligns with upregulation of CTL signature genes and B cell markers, resulting in parasite clearance and protection (Braden et al., 2018).

### 4.4 A strong intestinal IgT^+^ B cell response leads protection in the sympatric fish

B cell and antibody responses stimulated upon T cell activation are the next adaptive immune effectors in the battle against myxozoan diseases. The mucosal B cell response was the most significant differential trait we observed between steelhead trout strains. Among the B cell subtypes we analyzed, the IgT response was the dominant one in the intestine. An intense IgT^+^ B cell response, spread along the intestinal mucosa and the lamina propria, was triggered in SYM fish, following a strong T cell response, whereas ALLO fish presented a mild and delayed IgT^+^ B cell response. In contrast, IgD^+^ B cells were almost absent in the intestine of control and exposed steelhead trout. Further investigation is warranted to decipher if those are double IgM^+^IgD^+^ B cells or if single IgD^+^ IgM^-^ B cells are involved in the immune response against *C. shasta* G0. Nonetheless, IgM^+^IgD^+^ B cells are rare in rainbow trout mucosal tissues, with most IgM^+^ B cells lacking IgD expression (Herranz-Jusdado et al., 2023). In any case, this study has evidenced immunologic traits of the SYM fish phenotype (early T cell activation and IgT^+^ cell recruitment), which might help to further establish markers for the selection of resistant fish stocks (e.g., *zap70, igt* expression).

### 4.5 Specific serum IgM appears late and does not differ among fish strains

By contrast, the specific circulating IgM response detected at 57 and 183 dpe was similar among SYM and ALLO steelhead. Thus, the lower parasite load in the intestines of SYM fish may not be attributed to the IgM response, whose contribution to host protection at the later time points seems limited. The strongest immunogenic labeling of *C. shasta* G0 was observed in trophozoites migrating through the intestinal layers and in luminal trophozoites, with high cell polarization and with pseudopodia that appeared involved in mucosal attachment. Interestingly, disporoblasts and mature spores seemed to lack immunogenicity, at least by IgM. While the specific IgT in sera was below detection threshold, we detected IgT^+^ immunolabel coating luminal parasite stages in both SYM and ALLO hosts, supporting its limited systemic involvement against this parasite and the important mucosal role of IgT. However, it has been suggested that antigens in *C. shasta* trophozoites may mimic trout antigens to evade immune recognition (Bartholomew et al., 1989), and we cannot exclude the possibility that the observed IgT coating on *C. shasta* G0 intestinal stages is rather a sign of parasite masking than of immune targeting.

Chronic or prolonged *C. shasta* infections provide the time frame for hosts to develop an Ig response, though to what extent those antibodies provide protection is still unclear (Bartholomew et al., 2022). Yet, the mucosal role of IgT was first reported from rainbow trout intestines infected with *C. shasta* G0 (Zhang et al., 2010). Supporting our results, an increased transcription of secreted *igm* and *igt* was triggered at 29 dpe to *C. shasta* G0 in rainbow trout intestines, and this upregulation was significantly higher for *igt* than for *igm* (Taggart-Murphy et al., 2021). SYM steelhead were capable of reverting enteritis and cleared *C. shasta* GIIR from the intestinal tissue by 60 dpe (Barrett et al., 2021). These fish evidenced at 21 dpe a strong induction of genes related to epithelial repair and to the adaptive immune response, including a notable upregulation of Ig transcription, particularly IgT. In general, acute *C. shasta* infections with the virulent genotypes GIIR in steelhead and chinook salmon (Hurst et al., 2019; Barrett and Bartholomew, 2021) or GI in chinook salmon (Bjork et al., 2014) induce *igm* and *igt* expression, although susceptible ALLO fish were not able to resolve infection or parasite proliferation and the inflammatory process continued. The adaptive response appears rather late and ineffective, as mortality due to fatal enteronecrosis occurs around 20 dpe. Increased Ig expression and recruitment of Ig^+^ B cells to the infection site is not uncommon among myxozoan infections, pointing to the importance of the specific local immune response. This has been reported from both intestinal infection models, *E. leei* in gilthead seabream and *E. scophthalmi* in turbot (Bermúdez et al., 2006; Estensoro et al., 2012; Robledo et al., 2014; Piazzon et al., 2016; Ronza et al., 2016). Parasite-specific circulating IgM was related to protection against reinfection in both enteromyxoses (Sitjà-Bobadilla et al., 2007; Picard-Sánchez et al., 2019). Yet, protective antibodies appear late after the primary challenge of naïve fish, and in the case of the more virulent and acute turbot enteromyxosis, specific IgM prevalence in serum is low and fish mortality is high (Sitjà-Bobadilla et al., 2006, 2007). The slower progression of the chronic and less virulent enteromyxosis in gilthead seabream, however, seems to provide the time frame for fish to build up a more effective adaptive response, which might be a scenario closer to G0 ceratomyxosis studied herein. Thus, as in gilthead seabream, it will be worth studying, through secondary challenges and passive immunization, whether the more effective primary response which culminates with higher titers of parasite-specific IgM protects steelhead against reinfection by *C. shasta* G0 or, furthermore, if it provides cross-protection against *C. shasta* GIIR. The role played by resistance traits related to T cell response and IgT^+^ B cell response in such potential cross-protection against other parasite genotypes upon a secondary challenge would also be worth studying.

Immunomodulatory evasion strategies, common among myxozoans, include increased Ig titers during an ineffective and delayed specific immune response along with B cell exhaustion (reviewed in Sitjà-Bobadilla (2008)). In fact, the expression of high Ig clonotype diversity upon polyclonal expansion of diverse Ig B cell subsets (hyperimmunoglobulinemia) was reported from the Ig repertoires of rainbow trout during the profound B cell dysregulation caused by *Tetracapsuloides bryosalmonae* kidney infection (Abos et al., 2018) and during *E. leei* intestinal infection in naïve gilthead seabream (Picard-Sánchez et al., 2020). Also, antigen expression shift and epitope masking might be used by myxozoans to evade recognition by specific antibodies (Holzer et al., 2021). Thus, it is still unclear whether the specific antibody responses against *C. shasta* G0 are immunoprotective and whether a differential clonal expansion among the Ig repertoire is induced in SYM and ALLO fish upon ceratomyxosis.

### 4.6 The ultrastructure of *C. shasta* G0 primary cells suggests functional specialization for migration, environment exploration, and nutrient acquisition

*C. shasta* G0 ultrastructure in intestinal sections revealed extracellular trophozoites in the paracellular space between enterocytes and in the intestinal lumen. Cell protrusions (pseudopodia) of different lengths were observed in primary cells of both, intratissue and free luminal trophozoites, reaching between host cells. Such pseudopodia are compatible with the ones reported by Alama-Bermejo et al. (2019) from *C. shasta* GIIR, which were associated with the parasite’s exploratory behavior in complex environments. Although *C. shasta* G0 ultrastructure was not studied by those authors, from the expression of motility-related factors, it was inferred that early *C. shasta* G0 intestinal stages were less active but performed strong directional and adhesive migration characterized by an elongated cell shape with a leading edge. We also observed such elongated parasite shapes in the intratissue stages by TEM and light microscopy. After the initial intestinal invasion, *C. shasta* G0 stages abandon migration by 29 dpe (Alama-Bermejo et al., 2019), which aligns with the time point at which we observed luminal parasite stages peaking. Primary cell folds and protrusions also increase the parasites’ absorptive surface, aiding nutrition from the host cells. Hence, pinocytotic vesicles for nutrient acquisition have been reported, and vacuoles, lysosomes, vesicles, and lipidic droplets in primary cells were interpreted as food reserves, which were scarce in secondary and tertiary daughter cells of *Enteromyxum* spp. (Redondo et al., 2003; Cuadrado et al., 2008). By TEM, we also observed numerous vesicles of different sizes and densities, and electrodense lipid inclusions in primary cells, indicative of their nourishment-related activity. By contrast, secondary cells contained a remarkable high abundance of mitochondria, RER, and ribosomes, indicative of their proliferative stage and high protein synthesis activity. Further, some of the infiltrated intraepithelial lymphocytes observed by TEM had an irregular contact surface with the parasite’s primary cell, which might be a sign of direct interaction between both cell types.

### 4.7 The response against *C. shasta* G0 in gills of both fish strains is succinct

Finally, the immune response in gills, the portal of entry for the parasite, deserves some attention. As soon as fish are exposed to the parasite, recognition events in both fish strains seem to trigger the T cell response, although activation seemed stronger in SYM steelhead, aligning with the observed earlier activation of the T cell response at the intestinal level. No significant differences were observed in regard to the scarce IgT^+^ B cells in the gills, and only SYM fish showed an increasing trend in IgT^+^ B cell numbers, which occurred immediately after parasite exposure and decreased over time. Interestingly, IgT immunolabel on the mucosal surface of gills was weak or null in ALLO fish. These observations may indicate the increased immunocompetence of SYM fish. Previous studies on the gill invasion and later dispersion of *C. shasta* stages have not reported histopathological findings during these early phases, and it was suggested that embedding of the early parasite stages in endothelial cells of the gill blood vessels might impede immune recognition (Bjork and Bartholomew, 2010). Also, parasite-induced immunosuppression aiding an initial host invasion was reported from the gills of SYM and ALLO steelhead at 1 dpe to *C. shasta* GIIR, which encompassed T cell development and activation (Barrett and Bartholomew, 2021). Thus, these previous results might depict a similar situation to the one occurring in host gills during *C. shasta* G0 invasion.

### 4.8 IgD^+^ B cells present an opposite response in gills and intestine of both fish strains

Surprisingly, although IgD^+^ B cells were the scarcest lymphocytes observed in gills, this was the only leukocyte type that increased significantly, and only in ALLO steelhead after parasite exposure. And more intriguingly, IgD^+^ B cells were almost absent in the intestine of ALLO, while they increased in SYM host. The specific role of this Ig class in defense remains an enigma. B cells co-expressing IgD and IgM are the most abundant B cell subset in fish systemic tissues such as blood or spleen. Nonetheless, B cell subsets exclusively expressing IgD (IgD^+^ IgM^-^ plasmablasts) have been identified in different mucosal surfaces, including intestine, gills, and skin (Castro et al., 2014; Perdiguero et al., 2019; Herranz-Jusdado et al., 2023). Furthermore, IgD seemed involved in maintenance of commensal microbiota homeostasis (Perdiguero et al., 2019), which may explain the IgD immunolabeling observed on the intestinal brush border. From our current results, we cannot attribute the observed IgD^+^ B cells to a specific cell subtype, but their increase after the parasite challenge seems to follow the common pattern of IgD responses against inflammatory stimuli in teleosts (Bjørgen et al., 2023; Herranz-Jusdado et al., 2023; Perdiguero et al., 2024; Karami et al., 2025).

## 5 Conclusions

The current comparative challenge of SYM and ALLO hosts with the non-pathogenic genotype *C. shasta* G0 provides clues to the differential parasite dynamics in the intestine (**Graphical Abstract**). In SYM steelhead trout, trophozoites proliferated less and sporogenesis was occasional. Histopathology revealed that intestinal hyperplasia was resolved in SYM fish, whereas it continued for more than 180 days in ALLO fish. The successful coordination and regulation of earlier T cell activation and a stronger subsequent mucosal IgT response in SYM host hindered luminal proliferation of the parasite and reversed the intestinal inflammation earlier than in ALLO fish. In contrast to the more virulent genotypes I and II, G0 seems not to induce excessive dysregulation of intestinal T and B cells, provokes a less severe disease pattern, and develops into a chronic sublethal mainly celozoic infection probably aided by the systemic IgM protection, even in ALLO fish. Furthermore, after the long host–parasite co-evolutionary history, the limited *C. shasta* G0 proliferation and sporogenesis in SYM hosts might point to an adjustment of the parasite’s life cycle, and the early and effective inflammatory and immune response in SYM intestines might point to a differential, more effective host strategy: both complement each other and would allow for greater parasite tolerance in the SYM fish hosts. The current results at tissue and protein levels will be validated by functional molecular studies, which will help establish resistance markers for disease control in fish stocks, and cross-protection studies with the distinct parasite genotypes will aid in the development of vaccination strategies. From a management perspective, stocking of ALLO fish into waters where G0 is present in the absence of the more virulent GII would likely result in parasite amplification and increased dissemination as a result of the longer period of parasite release. While this may not result in direct mortality of SYM hosts, these fish did show disease pathology and presumed reduced fitness before the infection resolved.

## Data Availability

The original contributions presented in the study are included in the article/**Supplementary Material**. Further inquiries can be directed to the corresponding author.
