# Supplementary material for: Better the devil you know: sympatric fish host copes better than allopatric with a myxozoan parasite
Source: Front Cell Infect Microbiol. 2025 Jun 27;15:1576014. doi: 10.3389/fcimb.2025.1576014 (PMC12245873; doi:10.3389/fcimb.2025.1576014)
Supplement: Supplementary file 1 [file DataSheet1.pdf]

Supplementary Material

Supplementary Table 1. Student *t*-tests comparing sympatric (SYM) vs allopatric (ALLO) fish within the control (unexposed) group for the different histopathological parameters and lymphocyte counts. Where no significant differences were found, data were pooled into a single control unexposed experimental group for the subsequent analyses. dpe = days post-exposure

Parameter	Compared experimental groups	dpe	Test	P value
submucosal thickness	control SYM vs control ALLO	8	t-test	0.155
		15		0.955
		22		0.054
		29		0.785
		57		0.787
		183		0.303
submucosal hyperplasia	control SYM vs control ALLO	8	t-test	0.143
		15		0.266
		22		0.769
		29		0.925
		57		0.135
		183		0.578
IELs	control SYM vs control ALLO	8	t-test	0.5
		15		0.35
		22		0.5
		29		0.5
		57		1.000
		183		0.333
Zap70+ T cells - INTESTINE	control SYM vs control ALLO	8	t-test	0.126
		15		0.502
		22		0.172
		29		0.372
		57		0.683
		183		0.708
IgT+ B cells - INTESTINE	control SYM vs control ALLO	8	t-test	0.5
		15		1.000
		22		0.5
		29		0.5
		57		0.667
		183		0.523
IgD+ B cells - INTESTINE	control SYM vs control ALLO	8	t-test	1.000
		15		1.000
		22		1.000

		29		1.000
		57		1.000
		183		1.000
Zap70+ T cells - GILL	control SYM vs control ALLO	1	t-test	0.198
		8		0.111
IgT+ B cells - GILL	control SYM vs control ALLO	1	t-test	0.5
		8		0.776
IgD+ B cells - GILL	control SYM vs control ALLO	1	t-test	1.000
		8		1.000

Supplementary Table 2. *Ceratonova shasta* intestinal parasite counts (mean ± SD) in exposed sympatric and allopatric steelhead trout along the parasite challenge. dpe = days post exposure

	**SYMPATRIC**			**ALLOPATRIC**		
	**Lamina propria trophozoites**	**Luminal trophozoites**	**Luminal disporoblasts**	**Lamina propria trophozoites**	**Luminal trophozoites**	**Luminal disporoblasts**
**8 dpe**	0 ± 0	0 ± 0	0 ± 0	0 ± 0	0 ± 0	0 ± 0
**15 dpe**	2.25 ± 2.63	2.50 ± 1.73	0 ± 0	1.50 ± 1.73	1.50 ± 1.29	0 ± 0
**22 dpe**	8.50 ± 5.07	66.25 ± 15.59	0 ± 0	2.00 ± 1.41	78.25 ± 35.43	0 ± 0
**29 dpe**	2.75 ± 0.50	105.50 ± 38.38	1.00 ± 1.16	9.75 ± 4.57	184.00 ± 88.37	115.25 ± 83.48
**57 dpe**	0.25 ± 0.50	86.25 ± 65.03	0 ± 0	3.25 ± 2.50	81.50 ± 10.47	3.50 ± 7.00
**187 dpe**	0 ± 0	45.75 ± 17.35	0 ± 0	1.75 ± 2.87	153.75 ± 82.36	16.00 ± 26.75

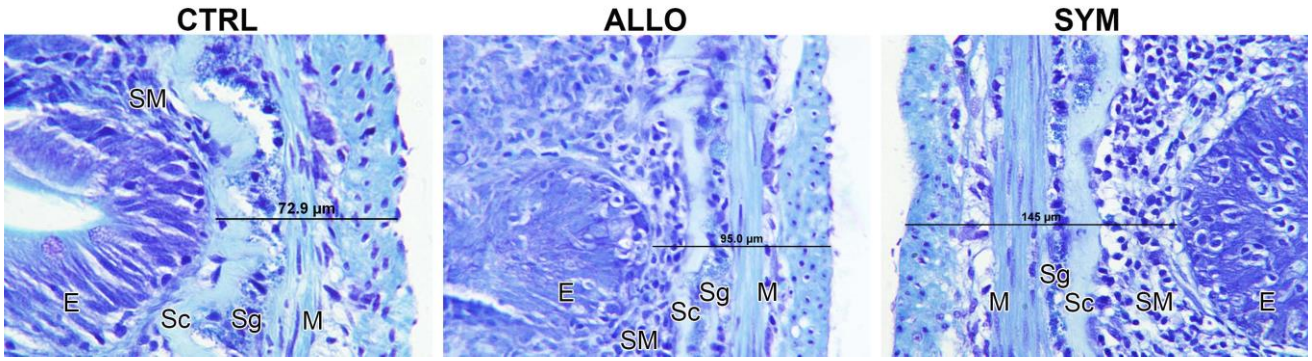

Supplementary Figure 1. Intestinal thickness measurements in control unexposed (CTRL) and *C. shasta* exposed allopatric (ALLO) and SYM (sympatric) steelhead trout strains. Giemsa stained paraffin sections. The different intestinal layers depicted from the internal luminal site to the most external are: epithelium (E), lamina propria-submucosa (SM), stratum compactum (Sc), stratum granulosum (Sg), muscularis (M).

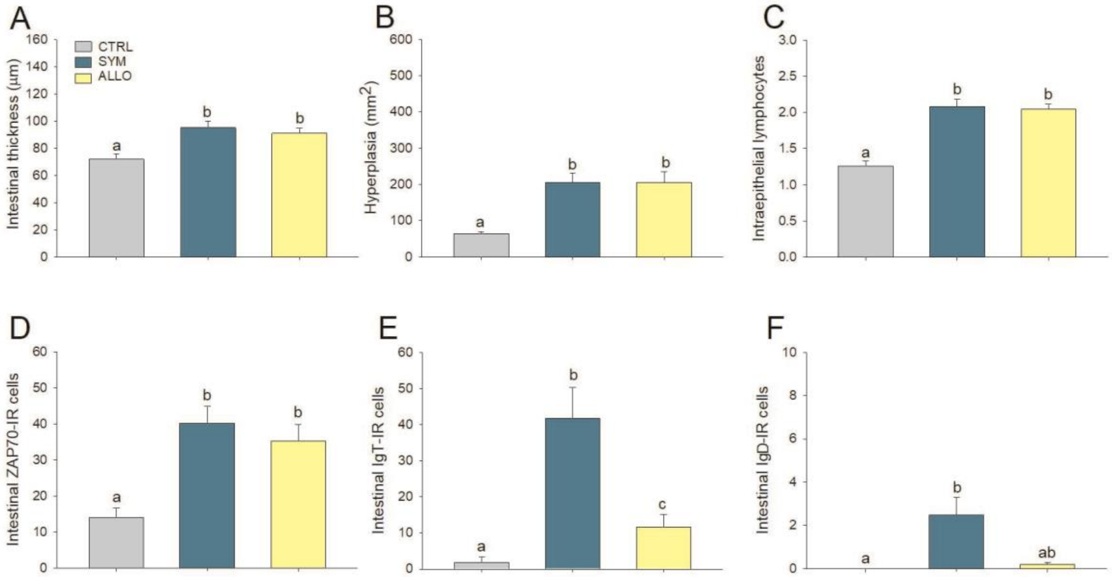

Supplementary Figure 2. Pooled data of the different sampling times in control unexposed (CTRL), and *Ceratonova shasta* exposed sympatric (SYM) and allopatric (ALLO) steelhead trout for the intestinal histopathological alterations: A) intestinal thickness, B) hyperplasia of the lamina propria-submucosa, C) lymphocyte epithelial infiltration scored from 0 (absence) to 3 (very abundant, meaning 25-30 intraepithelial lymphocytes per microscope field at 500x magnification); and for intestinal immunoreactive (IR) lymphocyte counts: D) Zap70-IR T cells, E) IgT-IR B cells, F) IgD-IR B cells. Different lowercase letters stand for significant differences between experimental groups. $P < 0.05$.

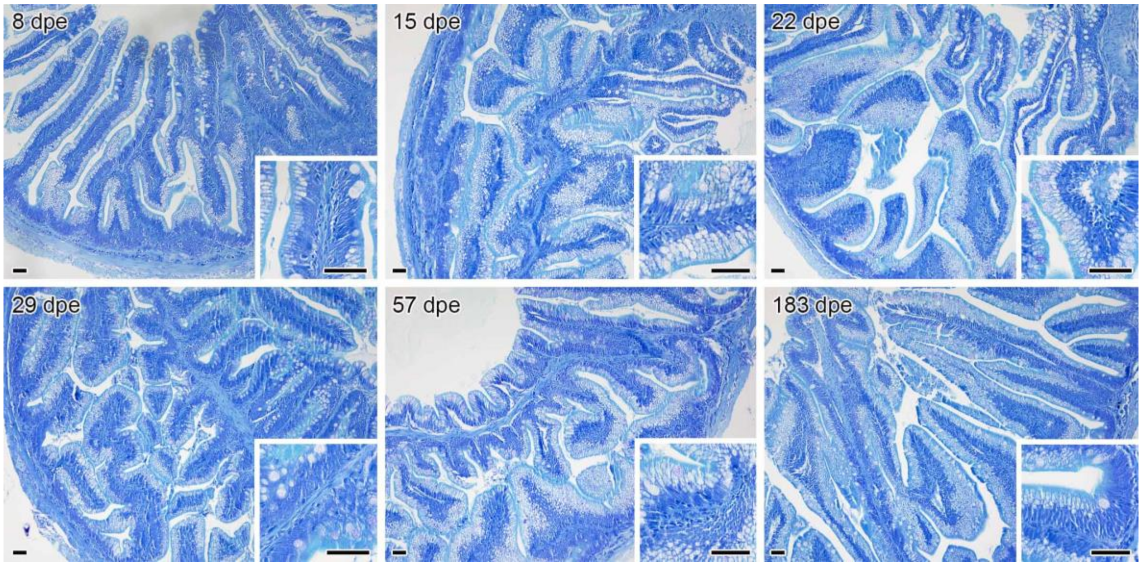

Supplementary Figure 3. Intestinal microphotographs of control unexposed allopatric (8 days postexposure (dpe), 15 dpe, 22 dpe) and sympatric (29 dpe, 57 dpe, 183 dpe) steelhead trout. Scale bars = 50 µm.

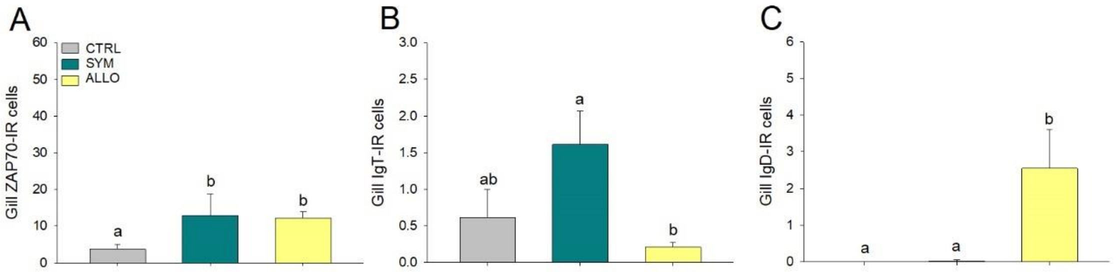

Supplementary Figure 4. Pooled data of the different sampling times in control unexposed (CTRL), and *Ceratonova shasta* exposed sympatric (SYM) and allopatric (ALLO) steelhead trout for the gill immunoreactive (IR) lymphocyte counts: A) Zap70-IR T cells, B) IgT-IR B cells, C) IgD-IR B cells. Different lowercase letters stand for significant differences between experimental groups. $P < 0.05$.
